# Romo1 Inhibition Induces TRAIL-Mediated Apoptosis in Colorectal Cancer

**DOI:** 10.3390/cancers12092358

**Published:** 2020-08-20

**Authors:** Min Jee Jo, Bu Gyeom Kim, Seong Hye Park, Hong Jun Kim, Soyeon Jeong, Bo Ram Kim, Jung Lim Kim, Yoo Jin Na, Yoon A. Jeong, Hye Kyeong Yun, Dae Yeong Kim, Jeongsu Han, Jun Young Heo, Young Do Yoo, Dae-Hee Lee, Sang Cheul Oh

**Affiliations:** 1 Department of Oncology, Korea University Guro Hospital, Seoul 08308, Korea; minjeeyoyo@nate.com (M.J.J.); qnrua10047@naver.com (B.G.K.); psh3938@hanmail.net (S.H.P.); jensyj85@gmail.com (S.J.); ilovewish777@naver.com (B.R.K.); clickkjl@naver.com (J.L.K.); wing1278@naver.com (Y.J.N.); leomi2614@naver.com (Y.A.J.); katecoco@hanmail.net (H.K.Y.); derrickdyblue22@gmail.com (D.Y.K.); 2 Graduate School of Medicine, Korea University College of Medicine, Seoul 02842, Korea; xpassion84@naver.com; 3 Department of Medical Science, School of Medicine, Chung-nam National University, Daejeon 35015, Korea; jeongsu_h@naver.com (J.H.); junyoung3@gmail.com (J.Y.H.); 4 Laboratory of Molecular Cell Biology, Graduate School of Medicine, Korea University College of Medicine, Korea University, Seoul 02842, Korea; ydy1130@korea.ac.kr; 5 Department of Marine Food Science and Technology, Gangneung-Wonju National University, Gangneung 25457, Korea

**Keywords:** reactive oxygen species modulator-1, tumor necrosis factor-related apoptosis-inducing ligand, Bax, Parkin, mitochondrial dysfunction

## Abstract

Tumor necrosis factor-related apoptosis-inducing ligand (TRAIL) is known to behave as an attractive anti-cancer agent in various cancers. Despite its promise TRAIL has limitations such as short half-life and rapid development of resistance. In this regard, approaches to sensitizers of TRAIL that can overcome the limitations of TRAIL are necessary. However, the molecular targets and mechanisms underlying sensitization to TRAIL-induced apoptosis are not fully understood. Here, we propose that reactive oxygen species modulator-1 (Romo1) as an attractive sensitizer of TRAIL. Romo1 is a mitochondrial inner membrane channel protein that controls reactive oxygen species (ROS) production, and its expression is highly upregulated in various cancers, including colorectal cancer. In the present study, we demonstrated that Romo1 inhibition significantly increased TRAIL-induced apoptosis of colorectal cancer cells, but not of normal colon cells. The combined effect of TRAIL and Romo1 inhibition was correlated with the activation of mitochondrial apoptosis pathways. Romo1 silencing elevated the protein levels of BCL-2-associated X protein (Bax) by downregulating the ubiquitin proteasome system (UPS). Romo1 inhibition downregulated the interaction between Bax and Parkin. Furthermore, Romo1 knockdown triggered the mitochondrial dysfunction and ROS generation. We validated the effect of combination in tumor xenograft model in vivo. In conclusion, our study demonstrates that Romo1 inhibition induces TRAIL-mediated apoptosis by identifying the novel mechanism associated with the Bax/Parkin interaction. We suggest that targeting of Romo1 is essential for the treatment of colorectal cancer and may be a new therapeutic approach in the future and contribute to the drug discovery.

## 1. Introduction

Colorectal cancer (CRC) is the third most common cancer and the fourth most common cause of death [1,2]. Despite the emergence of new treatments for colorectal cancer, new treatment options are still limited [1].

TNF-related apoptosis-inducing ligand (TRAIL) is a member of the tumor necrosis factor (TNF) family of ligands capable of causing apoptosis. TRAIL activates apoptosis in a wide range of cancer cells through interaction with the death receptors [3]. However, it does not show cytotoxicity in normal cells. In this regard, TRAIL is a promising new biological anticancer drug [4,5]. Despite the tumor-killing potential of TRAIL, TRAIL therapy has major limitations, namely a short half-life and the existence of TRAIL-resistant cancer populations. Therefore, elucidation of the molecular targets and signaling pathway responsible for TRAIL action is imperative for devising effective therapeutic strategies for overcome these limitations [6,7]. Moreover, it is important to develop a combination treatment strategy using a sensitizer that can increase the sensitivity of TRAIL.

Romo1, a novel mitochondrial protein, is the key regulator of mitochondrial ROS production and an essential factor for cancer cell proliferation and invasion [8]. It was observed that Romo1 is up-regulated in various cancers [9]. Previous studies have shown that Romo1 regulates ROS generation within cells and is involved in cellular processes such as cell proliferation, senescence, and death [8,10]. In addition, Romo1 is an essential regulator and act as a redox sensor in mitochondrial dynamics [11,12]. Therefore, we hypothesized that the sensitivity of TRAIL could be increased by targeting Romo1 to induce intrinsic apoptosis cascade associated with mitochondria. We focused on the role of Romo1 based on the result that Romo1 silencing disrupts the mitochondrial network by collapse of the function of mitochondria. This study showed that the novel role of Romo1 regulates the interaction between Bax and Parkin and controls the cellular events in an apoptotic cascade. However, signals and proteins modulate Romo1 activities and the association between mitochondrial dynamics and Romo1-induced apoptotic cascades are not well understood.

In our studies, we demonstrated whether Romo1 inhibition enhances the sensitivity of TRAIL in CRC. We found that Romo1 inhibition increased TRAIL-mediated apoptosis by elevating Bax expression. Moreover, Romo1 inhibition promoted apoptosis by disrupting mitochondrial function and generating ROS. Taken together, we propose that targeting Romo1 can be a sensitizer of TRAIL and it may be a new therapeutic strategy for cancer.

## 2. Results

### 2.1. Romo1 Inhibition Enhances TRAIL Sensitivity in CRC

**Figure 1:**
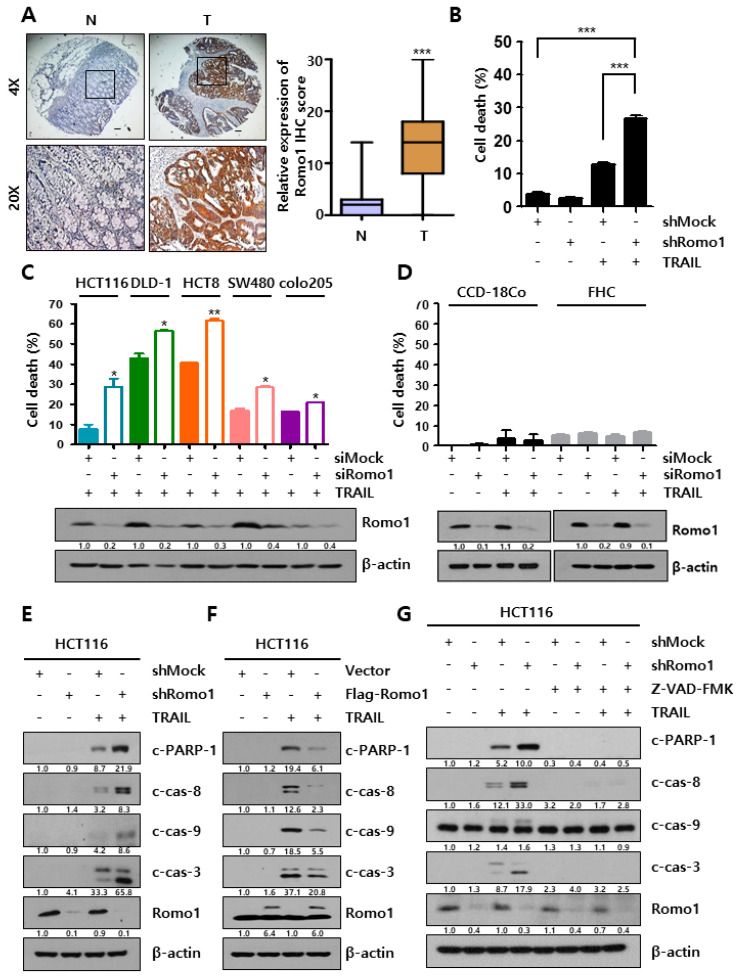
Effects Ablation of Romo1 enhances TRAIL-induced apoptosis. (**A**) Representative images of immunohistochemical analysis on Romo1 expression in human normal and colon cancer tissues. (Scale bar 200 μm and 50 μm). Box plots of the immunohistochemical staining scores of Romo1 in the normal (*n* = 54) and colon cancer (*n* = 190) tissues. (**B**) Cell death of HCT116 stable cells with TRAIL was analyzed by performing flow cytometry. (**C**) Flow cytometry analysis of HCT116, DLD-1, HCT8, SW480, and colo205 cells were transfected with control siRNA and Romo1 siRNA. TRAIL was added to Transfected cells for 6 h and apoptosis was determined by performing Annexin V/PI staining. Expression of Romo1 protein were detected by western blot analysis. (**D**) Human normal colon cell lines CCD-18Co and FHC cells were transfected with Romo1 siRNA. Transfected cells were treated with 5 ng/mL of TRAIL for 6 h. Cell death was analyzed by performing flow cytometry. Romo1 protein were measured using western blot analysis. (**E**) TRAIL treated or untreated stable cells were harvested for performing immunoblotting. (**F**) HCT116 cells were transfected with empty vector and Flag-Romo1 plasmid after then, treated with TRAIL for 6 h. protein expression were measured by immunoblotting. (**G**) Stable cells were pre-treated with 25 μM of Z-VAD-FMK for 1 h. After pre-treatment, stable cells were treated with TRAIL for 6 h. Protein expression were detected by western blot. The Quantification of blots with statistics of Figure 1C are shown in Figure S7. The Quantification of blots with statistics of Figure 1D are shown in Figure S8. The Quantification of blots with statistics of Figure 1E are shown in Figure S9. The Quantification of blots with statistics of Figure 1F are shown in Figure S10. The Quantification of blots with statistics of Figure 1G are shown in Figure S11. *, **, *** stands for * *p* < 0.05, ** *p* < 0.01, and *** *p* < 0.001. The Whole Blots for Western Blot analysis for Figure 1C are shown in Figure S23. The Whole Blots for Western Blot analysis for Figure 1D are shown in Figure S24. The Whole Blots for Western Blot analysis for Figure 1E are shown in Figure S25. The Whole Blots for Western Blot analysis for Figure 1F are shown in Figure S26. The Whole Blots for Western Blot analysis for Figure 1G are shown in Figure S27.

To explore the role of Romo1 in CRC, immunohistochemistry (IHC) staining was performed on tumor microarray (TMA) slides containing colorectal cancer tissues and normal colon tissue samples. According to the IHC staining results, we observed high expression level of Romo1 in CRC tissues (Figure 1A). Moreover, the expression of endogenous Romo1 was elevated in colorectal cancer cell lines comparing normal colon cell lines (Figure S1). To investigate the function of Romo1 on TRAIL-mediated apoptosis, HCT116 shMock and shRomo1 stable cells were treated with TRAIL. We found that the TRAIL-mediated cell death was remarkably increased in Romo1 knockdown (Figure 1B).

In addition, the cell images showed the morphologic changes of TRAIL-mediated cell death (Figure S2). To further study cell death, Annexin V/PI staining was conducted in various CRC cell lines including HCT116, DLD-1, HCT8, SW480, and colo205 cells. Interestingly, TRAIL mediated cell death was increased in Romo1-silenced cells (Figure 1C). However, the effect of Romo1 on TRAIL mediated cell death was not observed in Human normal colon cells (CCD-18Co and FHC) (Figure 1D and Figure S3). Next, we confirmed the combined effects of TRAIL and Romo1 silencing on protein expression levels by performing western blot (Figure 1E). Expression of apoptosis biomarkers, such as c-PARP-1, c-caspase-8, c-caspase-9, and c-caspase-3 were highly increased in Romo1-silenced cells treated with TRAIL and cell death were significantly increased when Romo1 was knocked down (Figure S4). In contrast, the effects of TRAIL-mediated cell death were rescued by Romo1 overexpression (Figure 1F and Figure S5). Next, cells were pre-incubated with Z-VAD-FMK, which is a pan-caspase inhibitor, to confirm that this combination effect was caspase cascade-dependent (Figure 1G). TRAIL-mediated apoptosis was blocked by the caspase inhibitor (Figure S6), and these results demonstrate that Romo1 inhibition was able to promote TRAIL-induced apoptosis in CRC cells.

### 2.2. Romo1 Knockdown Decreases Bax Ubiquitination by Decreasing Interaction with Parkin

**Figure 2:**
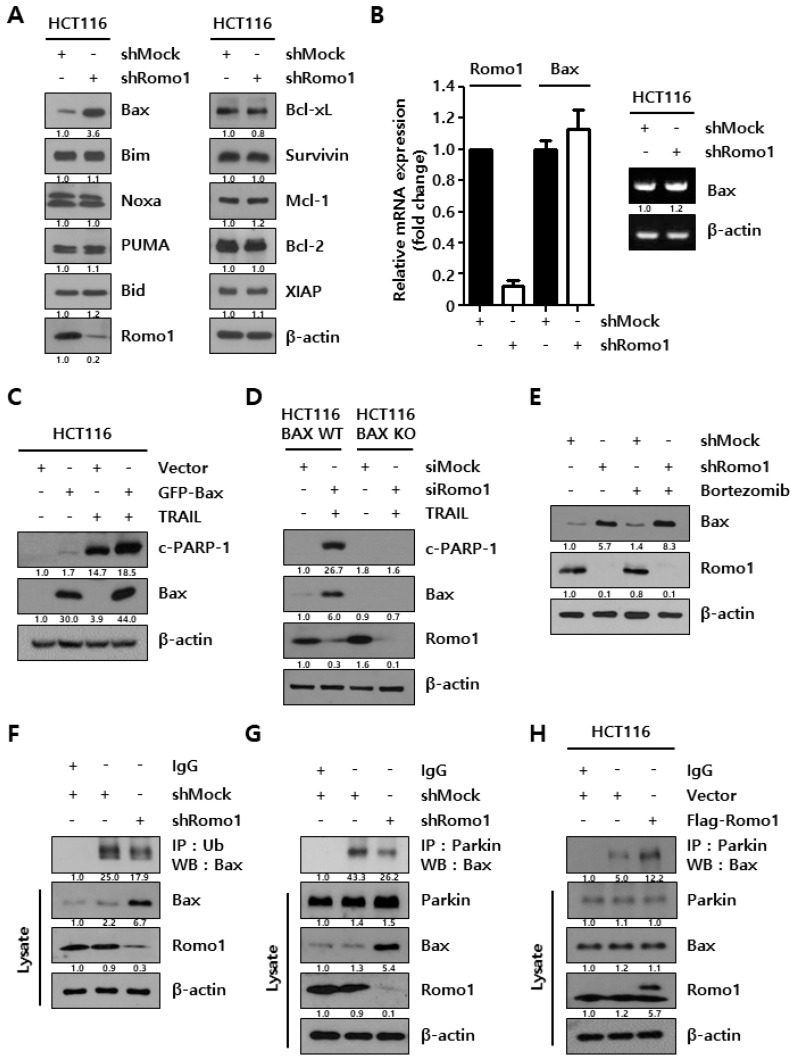
Romo1 silencing decreases Bax ubiquitination by decreasing the interaction Bax and Parkin. (**A**) HCT116 shMock and shRomo1 cells were harvested, and assessed by western blot. (**B**) Total RNA was isolated from HCT116 shMock and shRomo1 cells and mRNA expression level of Bax was assessed by performing RT-PCR and qRT-PCR. (**C**) HCT116 cells were transfected with empty vector and GFP-Bax plasmid. Transfected cells were treated with TRAIL for 6 h. Protein expression of c-PARP-1 and Bax were estimated by western blot. (**D**) HCT116 BAX WT and HCT116 BAX KO cells were transfected with control siRNA and Romo1 siRNA. Transfected cells were treated with 5 ng/mL TRAIL for 6 h. The protein levels of c-PARP-1, Bax, and Romo1 were detected by western blot. (**E**) Bax expression was determined through western blot after 10 nM bortezomib incubation for 24 h in both stable cells. (**F**) HCT116 shMock and shRomo1 stable cells were immunoprecipitated with the IgG or anti-Ub antibody and immunoblotted for Bax. (**G**) The interaction between parkin and Bax was analyzed by performing co-immunoprecipitation assay. Lysates of stable cells were immunoprecipitated with the IgG or Parkin antibody and immunoblotting was performed with Bax. (**H**) HCT116 cells were transfected with Flag-Romo1 plasmid. The interaction between parkin and Bax was analyzed by performing co-IP. Lysates were immunoprecipitated with the IgG or Parkin antibody and then immunoblotting was performed with Bax. The Quantification of blots with statistics of Figure 2A are shown in Figure S12. The Quantification of blots with statistics of Figure 2C are shown in Figure S13. The Quantification of blots with statistics of Figure 2D are shown in Figure S14. The Quantification of blots with statistics of Figure 2E are shown in Figure S15. The Quantification of blots with statistics of Figure 2F are shown in Figure S16. The Quantification of blots with statistics of Figure 2G are shown in Figure S17. The Quantification of blots with statistics of Figure 2H are shown in Figure S18. The Whole Blots for Western Blot analysis for Figure 2A are shown in Figure S28. The Whole Blots for Western Blot analysis for Figure 2C are shown in Figure S29. The Whole Blots for Western Blot analysis for Figure 2D are shown in Figure S30. The Whole Blots for Western Blot analysis for Figure 2E are shown in Figure S31. The Whole Blots for Western Blot analysis for Figure 2F are shown in Figure S32. The Whole Blots for Western Blot analysis for Figure 2G are shown in Figure S33. The Whole Blots for Western Blot analysis for Figure 2H are shown in Figure S34.

To determine the mechanism of action of Romo1 silencing, we investigated the apoptotic protein levels under Romo1 knockdown conditions (Figure 2A). We found that the expression level of Bax protein was greatly elevated in Romo1 knockdown. Moreover, mRNA expression level of Bax was examined by performing RT-PCR and qRT-PCR (Figure 2B). As a result, significant difference was not observed at mRNA expression level of Bax. These data imply that Romo1 upregulated Bax at the post-translational level, not at the transcriptional level. To study the importance of Bax upregulation on apoptosis, cells were transfected with GFP-Bax plasmid and treated with TRAIL (Figure 2C). We observed that Bax overexpression promoted to sensitize TRAIL treatment. On the contrary, Romo1inhibition-induced TRAIL-mediated apoptosis was diminished in HCT116 Bax KO cells (Figure 2D). These results indicate that Bax regulation by Romo1 knockdown is a critical mediator of TRAIL-mediated apoptosis. To determine the mechanism underlying Romo1 knockdown-induced Bax, we investigated Bax protein level following the treatment of bortezomib, a proteasome inhibitor, in HCT116 shMock and HCT116 shRomo1 stable cells (Figure 2E). Bax protein level was increased in bortezomib-treated Romo1-silenced cells, suggesting that Romo1 knockdown promotes Bax through ubiquitin-proteasome system (UPS). To verify Bax ubiquitination, co-immunoprecipitation (Co-IP) assay was performed in Romo1-silenced cells (Figure 2F). We found that Bax ubiquitination decreased upon Romo1 inhibition condition. Therefore, we attempted to identify the modulator of Bax ubiquitination. Parkin is an E3 ligase that ubiquitinates endogenous Bax [13]. Co-IP assay was conducted to investigate whether Romo1 inhibition regulates Bax ubiquitination through Parkin. The interaction between Bax and Parkin was significantly decreased in Romo1-silenced cells (Figure 2G).

As expected, the interaction between Bax and Parkin was increased in Romo1-overexpressing cells (Figure 2H). These results revealed that Romo1 inhibition decreases the ubiquitination of Bax by disrupting the interaction between Bax and its E3 ligase. These observations demonstrate that Romo1 contributes to Parkin-mediated ubiquitination of Bax.

### 2.3. Romo1 Inhibition Triggers Mitochondrial Dysfunction and Loss of MMP

**Figure 3:**
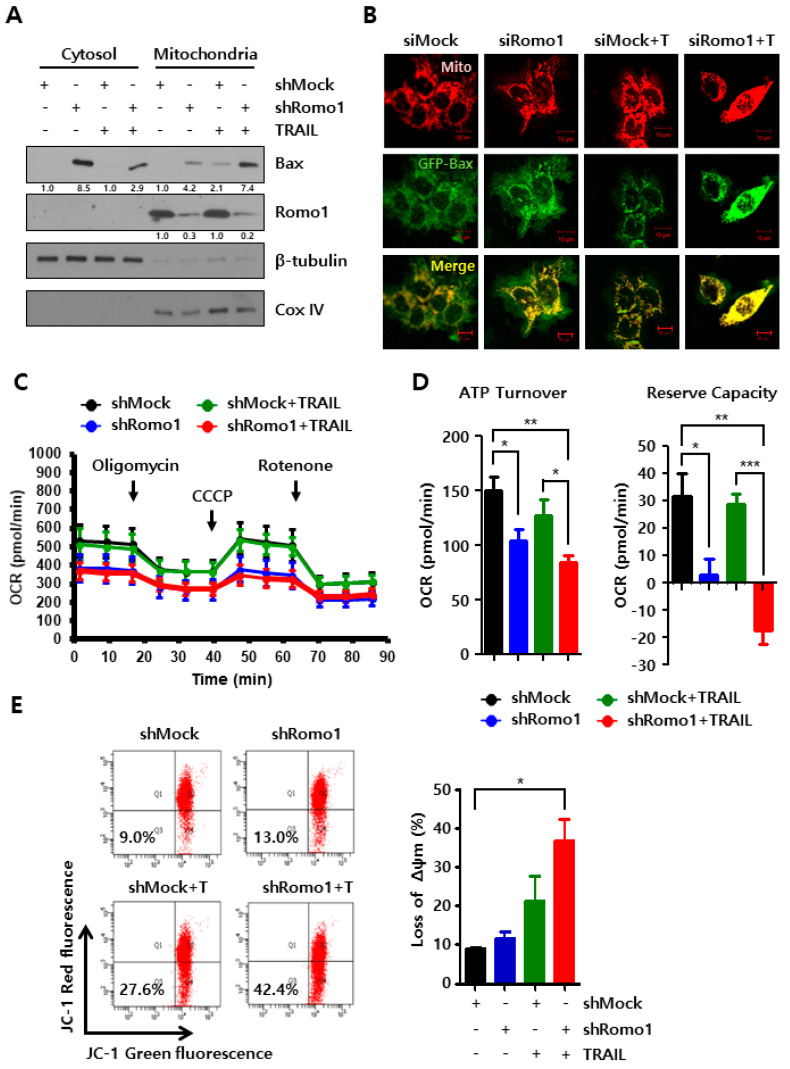
Romo1 inhibition causes mitochondrial dysfunction. (**A**) Cells were harvested and suspended in an isolation buffer. Cells were disrupted through homogenization, and obtained homogenates were seperated using centrifugation. The fraction of cytosol and mitochondria were assessed by immunoblotting. (**B**) HCT116 MitoRED cells were transfected with Romo1 siRNA and GFP-Bax plasmid. Immunofluorescence images showed the expression of Bax. (Scale bar, 10 μm) (**C**) OCR in stable cells treated or untreated with TRAIL for 4 h. OCR was measured by addiction of 2 μg/mL Oligomycin, 5 μM CCCP, and 2 μM Rotenone. (**D**) The bar graph of ATP Turnover OCR and Reserve Capacity were quantified using Seahorse Wave software. (**E**) TRAIL treated stable cells were stained with JC-1 dye. Loss of MMP (ΔΨm) was determined by executing flow cytometry. The bar graph represents the quantification of flow cytometry analysis. The Quantification of blots with statistics of Figure 3A are shown in Figure S19. *, **, *** stands for * *p* < 0.05, ** *p* < 0.01, *** *p* < 0.001. The Whole Blots for Western Blot analysis for Figure 3A are shown in Figure S35.

Romo1 is reported to closely associate with mitochondrial dynamics and Bax supports the mitochondrial network [12,14]. To determine whether Romo1 silencing affects the translocation of Bax, we analyzed the proteins present in the mitochondrial fraction (Figure 3A). We found that Bax translocation from the cytosol to the mitochondria increased in TRAIL-treated Romo1 knockdown cells. Immunofluorescence staining of MitoRed-treated HCT116 cells transfected with GFP-Bax plasmid revealed Bax expression within the mitochondria (Figure 3B). Furthermore, Bax expression increased in Romo1 silenced cells and Bax expression was highly elevated following TRAIL treatment. In this regard, we decided to explore mitochondrial function during Romo1 inhibition. Oxygen consumption rate (OCR) was estimated using the XF24 extracellular flux analyzer, and electron transport chain inhibitors, such as oligomycin (a complex V inhibitor), CCCP (a mitochondrial uncoupler), and rotenone (a complex III inhibitor) were used to examine mitochondrial dysfunction (Figure 3C). ATP turnover OCR was defined as the difference of value between basal and oligomycin-inhibited OCR. Reserve respiratory capacity was assessed as the difference of value between the basal and maximal OCR. ATP turnover OCR and reserve respiratory capacity greatly decreased in response to Romo1 inhibition (Figure 3D).

TRAIL treatment did not affect the OCR. Moreover, we performed JC-1 staining to investigate the loss of the mitochondrial membrane potential (MMP). JC-1 monomer emits green fluorescence at low MMP, while green fluorescent dye aggregates and emits red fluorescence at high MMP [15]. Flow cytometry data showed increased green fluorescence in Romo1-silenced cells with TRAIL treatment (Figure 3E). Quantitative data were evaluated with the Image J program. These results indicate that Romo1 inhibition leads to mitochondrial dysfunction and triggers collapse of MMP.

### 2.4. Mitochondrial Dysfunction upon Romo1 Inhibition Causes ROS

**Figure 4:**
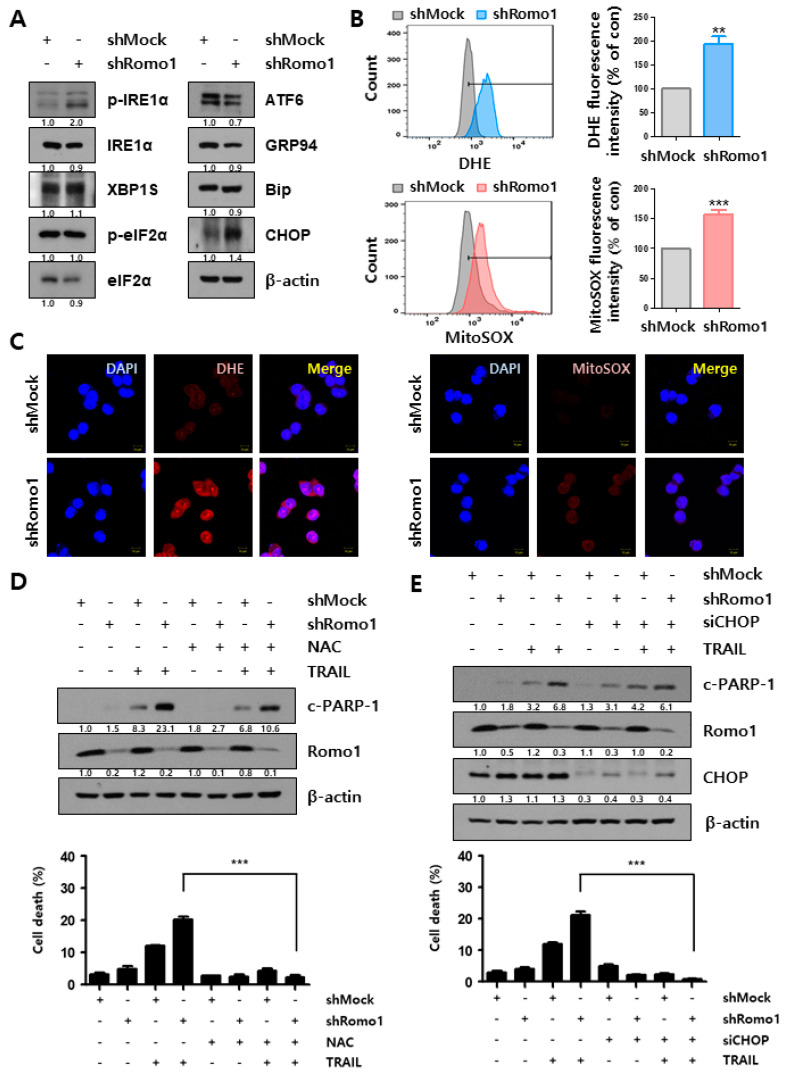
Collapse of mitochondrial function by Romo1 inhibition occurs ROS. (**A**) HCT116 shMock and shRomo1 cells were harvested and lysates were determined by western blotting. (**B**) HCT116 shMock and shRomo1 cells were dyed with 10 μM DHE or 5 μM MitoSOX for 30 min at 37 °C. Stained cells were evaluated using flow cytometry. The bar graph shows DHE and MitoSOX fluorescence intensity normalized to shMock. (**C**) HCT116 shMock and shRomo1 cells were dyed with DHE or MitoSOX for 30 min at 37 °C. Stained cells were evaluated using immunofluorescence staining. (Scale bar, 10 μm) (**D**) HCT116 shMock and shRomo1 stable cells were pre-treated with 5 μM NAC for 1 h. Pre-treated cells were treated with TRAIL and harvested for immunoblotting. Cell death was measured by flow cytometry. (**E**) HCT116 shMock and shRomo1 stable cells were transfected with control siRNA and CHOP siRNA. Transfected cells were treated with TRAIL for 6 h. The expression of c-PARP-1 and Romo1 were assessed by immunoblotting. Cell death was detected by flow cytometry. The Quantification of blots with statistics of Figure 4A are shown in Figure S20. The Quantification of blots with statistics of Figure 4D are shown in Figure S21. The Quantification of blots with statistics of Figure 4E are shown in Figure S22. **, *** stands for ** *p* < 0.01, and *** *p* < 0.001. The Whole Blots for Western Blot analysis for Figure 4A are shown in Figure S36. The Whole Blots for Western Blot analysis for Figure 4D are shown in Figure S37. The Whole Blots for Western Blot analysis for Figure 4E are shown in Figure S38.

The endoplasmic reticulum (ER)-stress is known to be functionally linked to the mitochondrial network [16]. ER-stress associated proteins were analyzed by western blot under Romo1 silencing (Figure 4A). As a result, the expression of DNA damage-inducible transcript 3 (CHOP), one of the ER-stress related proteins, was increased in Romo1-silencing conditions. Accumulating evidence suggests that ER-stress is strongly related to the level of reactive oxygen species (ROS) with redox signaling mediators [17]. We measured ROS level using flow cytometry and immunofluorescence staining (Figure 4B,C). Interestingly, we found that Romo1 knockdown increased ROS level. This result suggests that the mitochondrial dysfunction by Romo1 inhibition causes ROS generation. To verify this, immunoblotting was conducted to study the effect of NAC, a ROS scavenger, on TRAIL-induced apoptosis (Figure 4D). As shown, NAC treatment blocked the increase of TRAIL-associated apoptosis following Romo1 inhibition. We further confirmed the effect of CHOP inhibition on Romo1 inhibition-induced TRAIL-mediated apoptosis. As a result, increasing TRAIL-related apoptosis by Romo1 inhibition was reduced under CHOP silencing condition (Figure 4E). These results demonstrate that the collapse of mitochondrial network by Romo1 inhibition produced ROS.

### 2.5. Romo1 Inhibition Promotes TRAIL-Induced Apoptosis In Vivo

**Figure 5:**
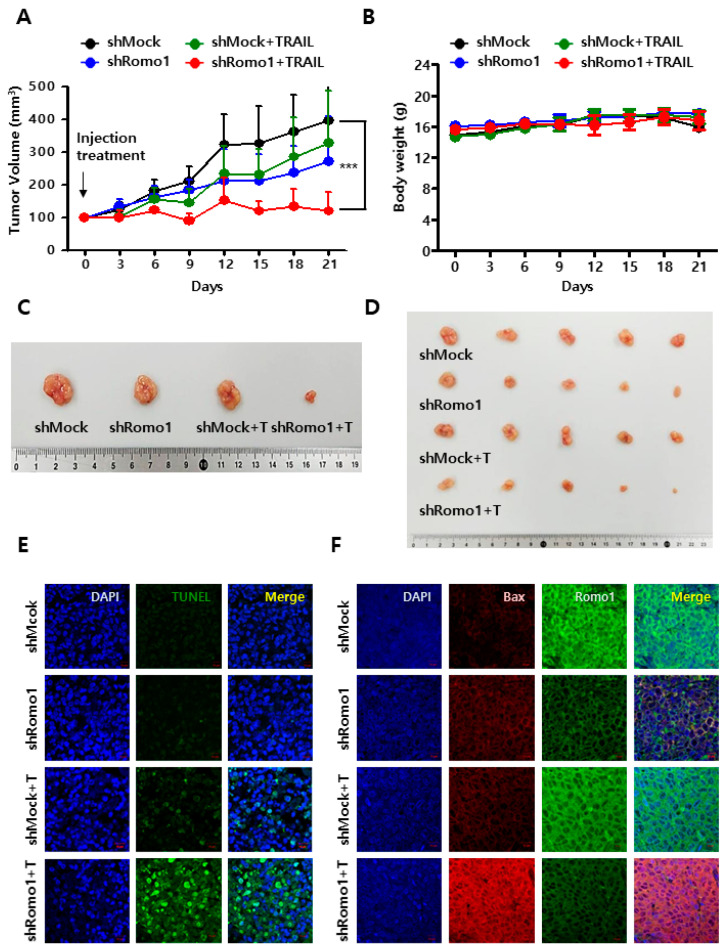
Effect of Romo1 suppression and TRAIL treatment on the tumor xenograft in vivo mouse model. (**A**,**B**) Effect of TRAIL treatment on the tumor xenograft in vivo mouse model established using BALB/c-nude mice injected subcutaneously with Romo1-deficient cells. Tumor growth (**A**) and body weight (**B**) after injecting shMock and shRomo1 HCT116 cell was monitored. (**C**,**D**) Representative images of tumor growth. (**E**) TUNEL assay in tumor tissues obtained from the xenograft mice. (Scale bar, 10 μm) (**F**) Sectioned tumor tissue was immunostained with the anti-Bax (red) and anti-Romo1 (green) antibodies. (Scale bar, 10 μm). *** stands for *** *p* < 0.001.

Based on the results of the in vitro experiments, we investigated the combined effect of TRAIL and Romo1 inhibition in tumor xenograft model in vivo. The tumor xenograft model was established by subcutaneous implantation of HCT116 shMock or shRomo1 stable cells into BALB/c-nude mice. After observing tumor formation, the mice were treated with or without TRAIL two times a week, and its effect on tumor growth was monitored for approximately 20 days. As shown in the data, combined treatment of TRAIL and Romo1 inhibition demonstrated anti-tumor effects compared with other groups (Figure 5A). The body weight of mice did not significantly change during the treatment period, and there were no treatment-related deaths (Figure 5B). The tumor mass size in TRAIL-treated Romo1-silenced group was significantly smaller compared to other groups (Figure 5C,D). We performed TUNEL assay of tumor tissues obtained from the mice to investigate whether Romo1 inhibition sensitizes TRAIL-induced apoptosis. We found that the apoptotic DNA fragmentation signal (green) was highly increased in TRAIL-treated Romo1-inhibited groups (Figure 5E). Bax expression levels in the tumor tissues obtained from mice were determined by performing the immunofluorescence assay (Figure 5F). As a result, the expression level of Bax was elevated in TRAIL-treated Romo1 knockdown groups. These results indicate that the combination of TRAIL and Romo1 inhibition had strong anti-tumor effects in vivo.

## 3. Discussion

Colorectal cancer is one of the most influential factors in cancer-related mortality [18,19]. Currently, colorectal cancer is in an area of high unmet need for effective new treatments. There are limited treatment options after second-line therapy [20,21,22]. Therefore, a new target agents and drug development should be pursued according to the situation.

TRAIL functions as an anticancer agent by inducing the apoptosis of tumor cells without affecting normal epithelial cells [4]. In this regard, TRAIL-based therapy merits attention for its promise as a cancer drug. However, a major concern of TRAIL therapy is that most tumor cells, including CRC cells, show TRAIL resistance and the short half-life in the clinical trials [23]. Various therapeutic strategies have been studied to overcome these limitations. However, the molecular targets and signaling pathways responsible for TRAIL action need to be fully elucidated [6]. Previous studies focused on the natural compounds, including curcumin and berberine, that may be used to treat various diseases by synergizing TRAIL [24,25]. Recent studies have examined new molecular targets as novel TRAIL-sensitizing agents. Possible targets for TRAIL therapy-enhancing activity are CHOP via p38/extracellular-signal-regulated kinase (ERK) MAPKs, and enhanced pro-apoptotic proteins, the c-Jun N-terminal kinases (JNKs) and nuclear factor kappa B (NF-kB) [26,27,28,29]. Resistance to anticancer drugs can be repressed by treatment with sensitizing agents that alter deregulated apoptosis signaling pathways in cancer cells. Several cancer models have been used to identify sensitizing agents [30,31].

In this study, we figured out that Romo1 inhibition enhanced the sensitivity of TRAIL in CRC cells by increasing Bax activation, mitochondrial dysfunction and ROS generation. We demonstrated that the role of Romo1 is essential for maintaining the mitochondrial dynamics as indicated by our OCR analysis and MMP assays. These findings may be associated with the deficient division of fuel and respiration in the inhibition of Romo1, accounting for the less-efficient performance of the electron transport chain and subsequent effect on ROS production. Moreover, MMP is important for maintaining the physiological function of the respiratory chain and for ATP production [32]. Mitochondrial dynamics going through fusion and fission are in charge of various mitochondrial functions including cell survival and death. A pathway of mitochondrial ROS production governed by Romo1 is known to be associated with the integrity of crista junction and OPA1 oligomerization [11]. After the inhibition of the electron transport chain, Bax can modulate cristae dynamics in dependence on changes of the mitochondrial redox state [33]. The results of our findings provide insights into how mitochondrial redox states leads to mitochondrial cristae splicing and mitochondrial network collapse in the context of cellular stress. Beyond the mechanisms responsible for Romo1 and Bax, future studies on mitochondrial crista conjugation and mitochondrial dynamics should be fully understand.

Romo1 is a mitochondrial inner membrane channel protein and a key factor of mitochondrial ROS. Romo1 has been reported to play a large role in cancer cell proliferation through myc, ERK activation and activation of NF-kB [27,34,35]. Moreover, myc, ERK and NFkB signaling are known to contribute to acquire resistance of TRAIL [36]. Here, in our study, it was identified as a mechanism related to the interaction between Bax and parkin, collapse of mitochondrial dynamics and ROS. Further studies on reported signaling related to Romo1 is needed.

The findings of our study highlight a mechanism by which Romo1-dependent ROS generation may regulate apoptosis through Bax activation in colorectal cancer. We present evidence that targeting the Romo1-Bax pathway can provide therapeutic benefits for treatment of colorectal cancer. Ulcerative Colitis (UC) represents an important risk factor for CRC and it is known that there is a high risk of colon cancer with ulcerative colitis [37,38,39]. As there is still no effective therapy for UC and UC-CRC, it is important to explore new drugs for UC and UC-CRC [40]. In this regard, the further studies of Romo1 involvement in CRC carcinogenesis with ulcerative colitis should be addressed. Furthermore, a new role of Romo1 as a sensitizer of TRAIL was identified in our study, but it will be necessary to investigate whether it affects apoptosis induced by chemotherapeutic reagents used in clinical application.

In summary, the findings of the present indicate that Romo1 inhibition promotes TRAIL-mediated apoptosis in CRC cells. The synergistic effect of elevated Bax activation induces collapse of MMP, releasing of cytochrome c, activation of caspases, thus deteriorating apoptosis of CRC cells. Our study suggests that targeting Romo1 may have strong anti-cancer effect as a sensitizer of TRAIL-based therapy and may contribute to improving the development of novel medicines for colorectal cancer.

## 4. Materials and Methods

### 4.1. Cell Culture

Human colon carcinoma cell lines DLD-1, SW480, HCT8, and colo205 were obtained from American Type Culture Collection (ATCC, Manassas, VA, USA) and grown in RPMI 1640 medium (Gibco, Grand Island, NY, USA) containing 10 % fetal bovine serum (FBS) and 100 mg/mL penicillin and streptomycin (P/S). Human colon carcinoma cell lines HCT116 and HCT116 Bax knockout (KO) cells were obtained from ATCC and maintained in Mccoy’s 5A medium (WELGENE, Gyeongsan, Korea) containing 10 % FBS and 100 mg/mL P/S. Human normal colon fibroblast cell lines CCD-18Co were purchased from ATCC and grown EMEM medium (ATCC) containing 10 % FBS and 100 mg/mL P/S. Human normal colon epithelial cell lines FHC were obtained from ATCC and maintained DMEM:F12 medium (Gibco) with supplementary according to the manufacturer’s instructions.

### 4.2. Transient and Stable Transfection

For transient transfection, CRC cells were transfected with expression plasmids using empty vector, GFP-Bax and Flag-Romo1 by using Lipofectamine 2000 reagent (Invitrogen, Carlsbad, CA, USA) and transfected with siRNA against control (sc-37007), Romo1 (sc-76423) and CHOP (sc-35437) by using Lipofectamine RNA iMAX (Invitrogen).

For stable transfection, stable Romo1-knockout cells were generated by transduction of lentivial plasmid vector for Romo1 shRNA (shRomo1) (sc-76423-SH) and control shRNA (sc-108060) purchased from Santa Cruz Biotechnology (Dallas, TX, USA). After lentiviral transduction on cell, cells were selected by puromycin at the concentration of 2 μg/mL. We performed clone screening and selection by western blot and qRT-PCR. Selected stable cells were grown and used in the experiment.

### 4.3. Reagents and Antibodies

Recombinant human TRAIL was purchased from R&D Systems (Minneapolis, MN, USA), and anti-Romo1 (TA505580) antibody was purchased from Origene (Rockville, MD, USA). Protein G PLUS-Agarose beads and anti-B-cell lymphoma 2 (BCL-2) (sc-509), anti-B-cell lymphoma-extra large (BCL-XL) (sc-8392), anti-ubiquitin (anti-Ub) (sc-9133), anti-parkin (sc-32282), anti-X-box binding protein 1S (XBP1S) (sc-8015), anti-Activating transcription factor 6 (ATF6) (sc-30597), and anti-GRP94 (sc-32249) antibodies were purchased from Santa Cruz Biotechnology. Anti-X-linked inhibitor of apoptosis protein (XIAP) (#2042), anti-cleaved Poly (ADP-ribose) polymerase (PARP) (#9541), anti-caspase 8 (#9496), anti-caspase 9 (#9509), anti-caspase 3 (#9662), anti-Bcl 2-like protein 11 (Bim) (#2933), anti-Noxa (#14766), anti-p53 upregulated modulator of apoptosis (PUMA) (#4976), anti-BH3 interacting-domain death agonist (Bid) (#2002), anti-IRE1α (#3294), anti-eIF2α (#9722), anti-phospho-eIF2α (#3597), anti-Binding immunoglobulin protein (Bip) (#3177), anti-DNA damage-inducible transcript 3 (CHOP) (#5554), anti-Bcl 2-associated X protein (Bax) (#7480), anti-cytochrome c oxidase IV (COX IV) (#4850), anti-survivin (#2808), and anti-Myeloid cell leukemia-1 (MCL-1) (#4572) antibodies were purchased from Cell Signaling Technology (Danvers, MA, USA). Anti-phospho-IRE1α (ab48187) was purchased from Abcam (Cambridge, MA, USA). Anti-β-tubulin (MMS-410P) antibody was purchased from Covance (Princeton, NJ, USA). Anti-β-actin (A5316) was purchased from Sigma Aldrich (St. Louis, MO, USA). Horseradish peroxidase-conjugated anti-mouse IgG and anti-rabbit IgG were purchased from Cell Signaling Technology.

### 4.4. Flow Cytometry Analysis

Cell apoptosis was estimated using Annexin V-FITC apoptosis detection kit (BioBud, Seoul, Korea). CRC cells were treated or untreated with 5 ng/mL TRAIL for 6 h. Next, cultured cells were trypsinized, centrifuged at 1800× *g* for 5 min. Cell pellet were suspended and dyed with Annexin V-FITC reagent and propidium iodide solution for 30 min at room temperature (RT) without light. Stained cells were evaluated by using flow cytometry.

Production of ROS was measured using dihydroethidium (DHE, Invitrogen) and MitoSOX RED (Invitrogen). For the assay, cells were treated or untreated with TRAIL for 6 h. And then cells were stained using 10 μM DHE or 5 μM MitoSox dye for 30 min in the dark at 37 °C. Next, stained cells were measured by flow cytometry and fluorescence intensity was used to quantify using FlowJo software (BD Biosciences, San Diego, CA, USA).

### 4.5. Reverse Transcription-Polymerase Chain Reaction (RT-PCR)

Total RNA was isolated from the cells by using TRIzol solution (Life Technologies, Carlsbad, CA, USA), according to the manufacturer’s instructions. Amplification of transcript was conducted using a reverse transcription-polymerase chain reaction kit (Life Technologies) and the following primers: Romo1 forward: 5′ CTG TCT CAG GAT CGG AAT GCG 3′, Romo1 reverse: 5′ CAT CGG ATG CCC ATC CAA TG 3′, Bax forward: 5′ ACC AAG AAG CTG AGC GAG TGT C 3′, Bax reverse: 5′ ACA AAG ATG GTC ACG GTC TGC C 3′, β-actin forward: 5′ ACC CAG ATC ATG TTT GAG AC 3′, and β-actin reverse: 5′ GGA GTT GAA GGT AGT TTC GT 3′.

### 4.6. Quantitative Real Time PCR (qRT-PCR)

qRT-PCR was performed using QuantStudio 6 Flex system and SYBR Green probes (Applied Biosystems, Foster City, CA, USA). Gene expression was normalized with the expression of glyceraldehyde-3-phosphate dehydrogenase (GAPDH) genes.

### 4.7. Immunoblotting

Immunoblotting was executed as previously described [8]. The protein expression was estimated using X-ray films and chemiluminescence protocol (DoGEN ECL; Daeil Lab Service Co. Ltd., Seoul, South Korea). Quantification of blots with statistics was determined using Image J program (NIH, Bethesda, MD, USA) and blots were normalized to β-actin.

### 4.8. Co-Immunoprecipitation (Co-IP)

Cell lysis was proceeded with 300 μL of lysis buffer containing 1 mM phenylmethylsulfonyl fluoride (PMSF) (Sigma), protease inhibitor, and phosphatase inhibitor (Cell Signaling Technology) on ice for 5 min. Following the lysis, cellular debris was removed by centrifugation. Protein concentration was determined by performing bicinchoninic acid (BCA) assay (Thermo Fisher Scientific, Waltham, MA, USA). Cells were quantified and incubated with the primary antibodies for overnight at 4 °C and followed by incubation with 30 μL of protein G PLUS-Agarose beads for 1 h at 4 °C. Immunoprecipitates were washed and separated by centrifugation at 15,000 rpm, and then heated with 2X sample buffer for immunoblotting.

### 4.9. The Mitochondria and Cytosol Fractionation

Cells were collected (approximately 2 × 10^7^ cells) and suspended in an isolation buffer solution [70 mM sucrose, 5 mM HEPES, 1 mM EGTA, 210 mM mannitol, phosphatase inhibitor and protease inhibitor]. Next, the homogenates by homogenizing were centrifuged at 17,000× *g* for 10 min. Obtained supernatant was used as a fraction of cytosol. Obtained pellet was resuspended with isolation buffer and used as a fraction of mitochondria.

### 4.10. Mitochondrial Membrane Potential (MMP) Measurement

MMP was measured through staining stable cells with JC-1 dye (Invitrogen). For this, the stable cells were dyed with JC-1 for 30 min at 37 °C. Next, the stained cells were collected and examined by using flow cytometry. The bar graph represents the quantification of loss of MMP (ΔΨm).

### 4.11. Oxygen Consumption Rate (OCR)

Stable CRC cells were seeded (density, 30,000 cells/well) in XF24 cell culture plates (V7-PS; Seahorse Bioscience, North Billerica, MA, USA) and treated with TRAIL for 4 h. 1 h before the measuring, the cultured medium was changed with glucose containing XF24 medium. The value of OCR was determined using XF24 extracellular flux analyzer. The measured OCR was validated by sequential addiction of 2 μg/mL of oligomycin (04876, Sigma Aldrich), 5 μM of carbonyl cyanide 3-chlorophenyl hydrazone (CCCP, C2759, Sigma Aldrich), and 2 μM of rotenone (R8875, Sigma Aldrich) in sequence. The parameters calculated OCR values were in accordance with Seahorse Wave software (Seahorse Bioscience). The calculation of parameter figured out according to this formula: ATP turnover (baseline respiration—oligomycin post injection respiration) and Reserve Capacity (CCCP stimulated respiration—baseline respiration).

### 4.12. Immunofluorescence Analysis

Stable cells were seeded above the cover slip and fixed with 3.7% formaldehyde for 15 min at RT and permiabilized with 0.5% Triton-X 100 in PBS for 15 min at RT. Then, the cells were incubated with a 3% BSA blocking buffer for 1 h at 4 °C. Following overnight incubation with the primary antibody at 4 °C. Cells were then incubated with Alexa Fluor 594-conjugated goat anti-rabbit secondary antibody (Invitrogen) or Alexa Fluor 488-conjugated goat anti-mouse secondary antibody (Invitrogen) at 4 °C for 17 min. After washing steps, the cover slip was mounted and images were visualized by a confocal microscope.

### 4.13. Immunohistochemical Analysis

Immunohistochemical analysis was conducted to evaluate Romo1 expression. After deparaffinization and antigen retrieval, tissue microarray (TMA) slides were incubated with 3% peroxide blocking reagent for 15 min and heated for 20 min at 100 °C. Next, the slides were universally blocked with a blocking buffer (Dako, Glostrup, Denmark). Following the incubation, slides were stained with anti-Romo1 antibody (dilution, 1:200). Slides not treated with the primary antibody were used as negative controls.

### 4.14. TUNEL Assay

Tissues from tumor were immersed with 4% paraformaldehyde and were embedded with paraffin. After deparaffinization, antigen retrieval, and blocking, the slides were dyed with an in situ cell death detection kit (Roche, Basel, Switzerland). DNA fragmentation associated apoptosis was examined by visualizing fluorescence images.

### 4.15. Tumor Xenograft In Vivo Model

All procedures of animal experiment were governed on the basis of animal care guidelines approved by Institutional Animal Care and Use Committee (IACUC) of Korea University College of Medicine (KOREA-2017-0103-C2). BALB/c-nude mice were taken from Orient Bio. HCT116 shMock or shRomo1 stable cells (density, 1 × 10^7^ cells/100 μL of PBS) were subcutaneously injected to the mice. After randomization, mice were monitored until their tumor size reached approximately 100 mm^3^ in size. Next, the mice were intraperitoneally injected with PBS or TRAIL (Koma Biotech, Seoul, South Korea) three times a week. The measurement of tumor size was performed every 2–3 days by using a caliper. The calculation of tumor volume figured out according to this formula: tumor volume (mm^3^) = W1 × W2 × W2/2 (where W1 and W2 are the largest and smallest tumor diameters, respectively).

### 4.16. Statistical Analysis

All experiments were fulfilled independently and confirmed at least three times. The significance of the statistics was determined based on the GraphPad InStat 6 Software (La Jolla, CA, USA). The results were expressed as the mean of arbitrary values ± standard error of the mean (SEM). All the results were evaluated by using unpaired Student’s *t*-test and *p*-value of less than 0.05 was considered significant. Each *, **, *** stands for * *p* < 0.05, ** *p* < 0.01, and *** *p* < 0.001.

## 5. Conclusions

**Figure 6:**
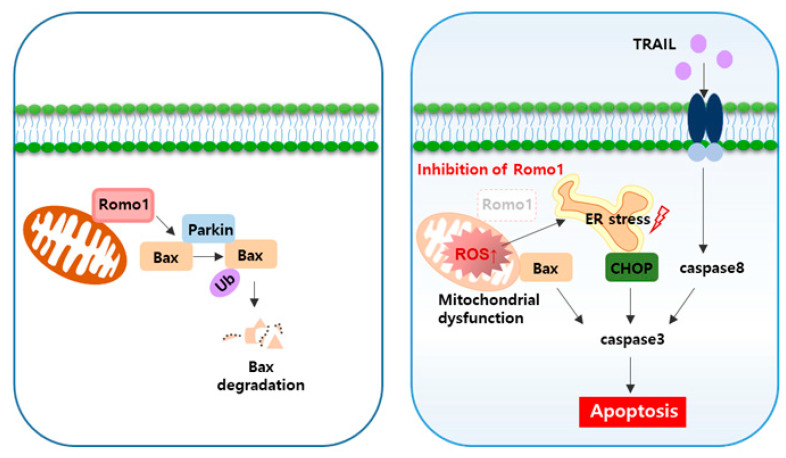
The TRAIL of oncogenes to apoptosis. A schematic model of the Romo1-associated TRAIL pathway.

We have demonstrated that Romo1 inhibition and TRAIL have a combined effect in colorectal cancer and identified the novel mechanism of this combined effect associated with the TRAIL pathway (Figure 6). In this paper, we suggest that targeting Romo1 is essential for the treatment of colorectal cancer and may be a new therapeutic strategy.

## Supplementary Materials

The following are available online at https://www.mdpi.com/2072-6694/12/9/2358/s1, Figure S1: Expression levels of Romo1 in human colorectal cancer cell lines, Figure S2: Morphologic changes of TRAIL-mediated cell death and trypan blue staining in HCT116 stable cells, Figure S3: TRAIL-mediated cell death with Romo1 overexpression in human normal colon cell lines, Figure S4: TRAIL-mediated cell death with Romo1 knock-down using Romo1 siRNA in HCT116 cells, Figure S5: TRAIL-mediated cell death with Romo1 overexpression in HCT116 cells, Figure S6: TRAIL-mediated cell death with Z-VAD-FMK in HCT116 stable cells, Figure S7–S22: Quantification of western blots with statistics, Figure S23–S40: Whole Blots for Western Blot analysis with molecular weight markers.
