# Supplementary material for: Romo1 Inhibition Induces TRAIL-Mediated Apoptosis in Colorectal Cancer
Source: Cancers (Basel). 2020 Aug 20;12(9):2358. doi: 10.3390/cancers12092358 (PMC7565722; doi:10.3390/cancers12092358)
Supplement: Supplementary file 1 [file cancers-12-02358-s001.pdf]

cancers

Supplementary Materials

Romo1 Inhibition Induced TRAIL-Mediated Apoptosis in Colorectal Cancer

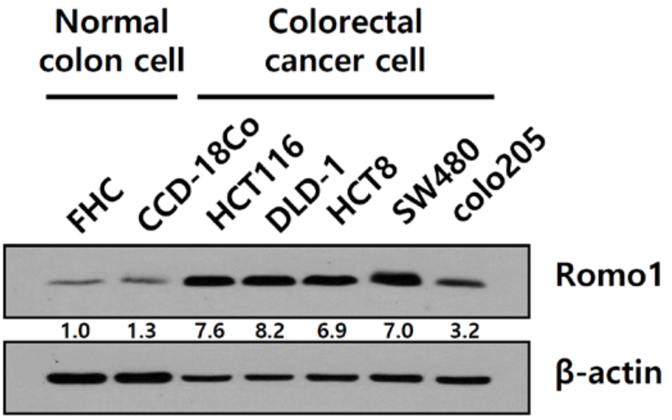

Figure S1. Expression levels of Romo1 in human colorectal cancer cell lines. Western blot analysis was performed by comparing the endogenous protein levels of Romo1 in five colorectal cancer cell lines (HCT116, DLD-1, HCT8, SW480and colo205) and normal colon cell lines (FHC and CCD-18Co). The Quantification of blots with statistics of Figure S1 are shown in Figure S39. The Whole Blots for Western Blot analysis for Figure S1 are shown in Figure S39.

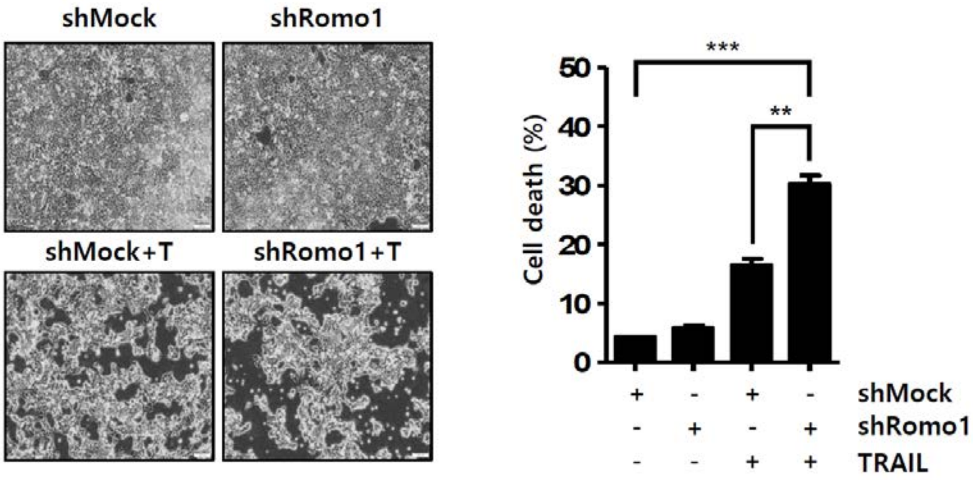

Figure S2. Morphologic changes of TRAIL-mediated cell death were measured by optical microscope (Left panel) (Scalebar, 100 μm). The cell death through trypan blue staining in HCT116 stable cells treated with 5ng/mL TRAIL for 6h (Right panel). **, *** stands for ** $p < 0.01$, and *** $p < 0.001$.

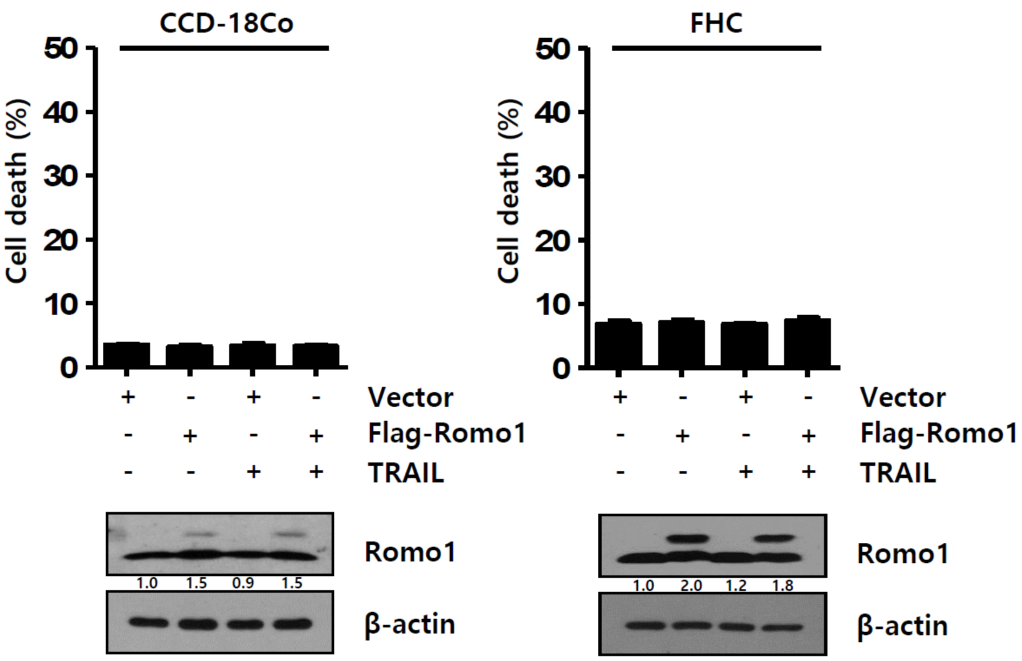

Figure S3. Normal colon cell lines (CCD-18Co and FHC) were transfected with empty vector or Flag-Romo1 plasmid. Transfected cells were treated with 5 ng/mL TRAIL for 6 h. Cell death was analyzed by flow cytometry. Expression of Romo1 was determined by performing western blot analysis. The Quantification of blots with statistics of Figure S3 are shown in Figure S40. The Whole Blots for Western Blot analysis for Figure S3 are shown in Figure S40.

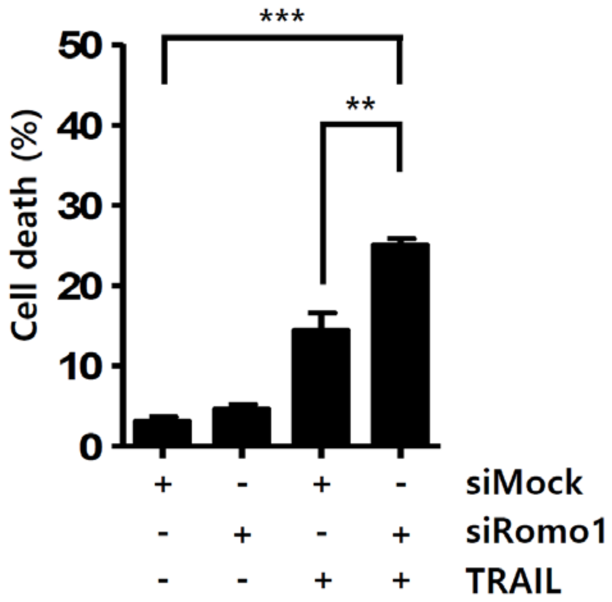

Figure S4. Cell death was measured by flow cytometry with Romo1 Knocked-down using Romo1 siRNA in HCT116 cells. **, *** stands for ** $p < 0.01$, and *** $p < 0.001$.

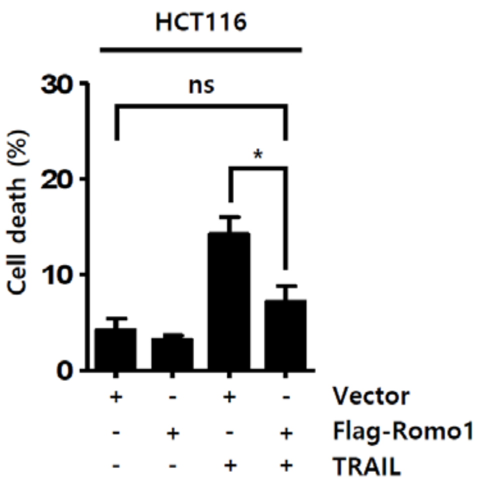

Figure S5. HCT116 cells were transfected with empty vector or Flag-Romo1 plasmid. Transfected cells were treated with 5 ng/mL TRAIL for 6 h. Cell death was analyzed by flow cytometry. * stands for * $p < 0.05$.

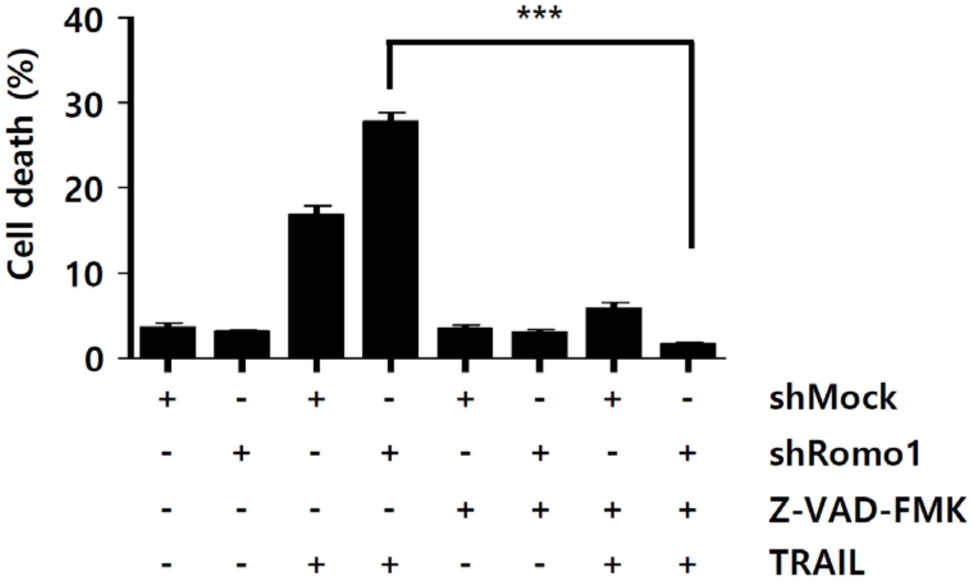

Figure S6. Cell death was analyzed by performing flow cytometry. HCT116 stable cells were pretreated with Z-VAD-FMK for 1 h. After then, cells were treated with 5 ng/mL of TRAIL for 6 h. *** stands for *** $p < 0.001$.

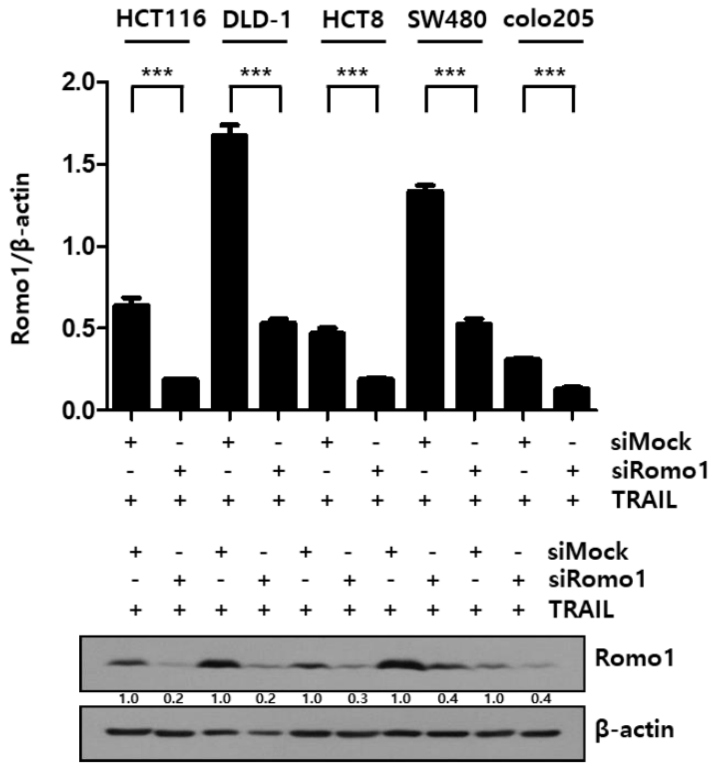

Figure S7. The bar graph represents the mean standard deviation (SD) of western blot band density of Figure 1C. *** stands for *** $p < 0\ 001$.

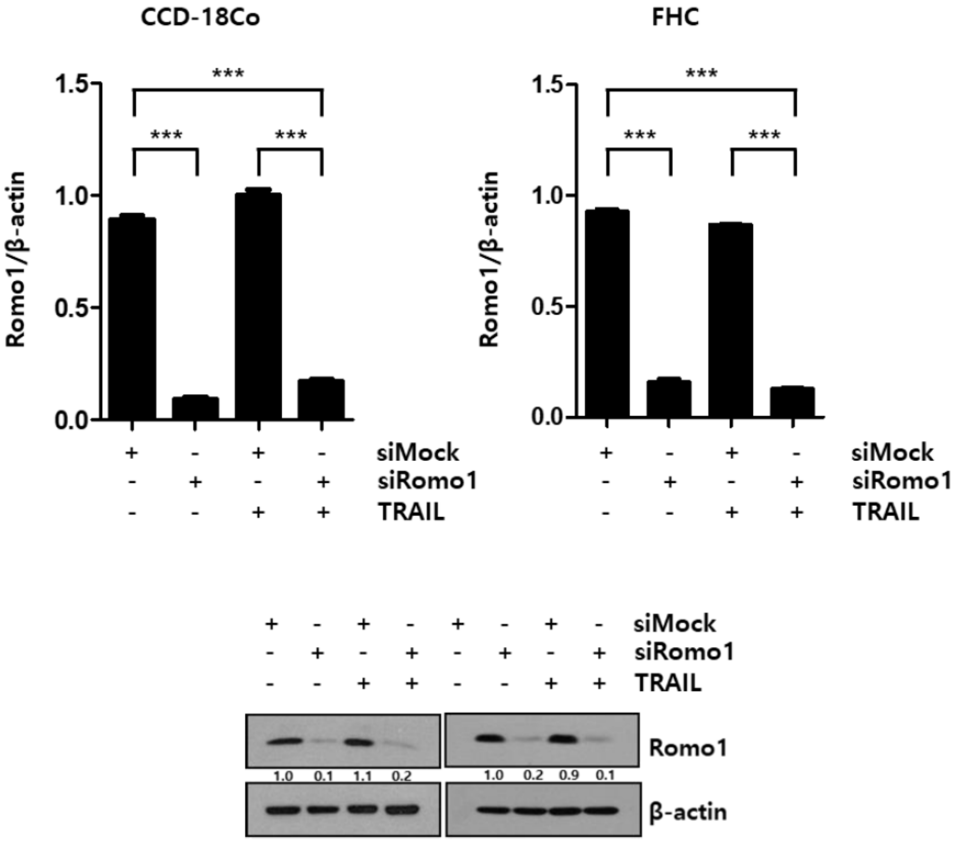

Figure S8. The bar graph represents the mean standard deviation (SD) of western blot band density of Figure 1D. *** stands for *** $p < 0.001$.

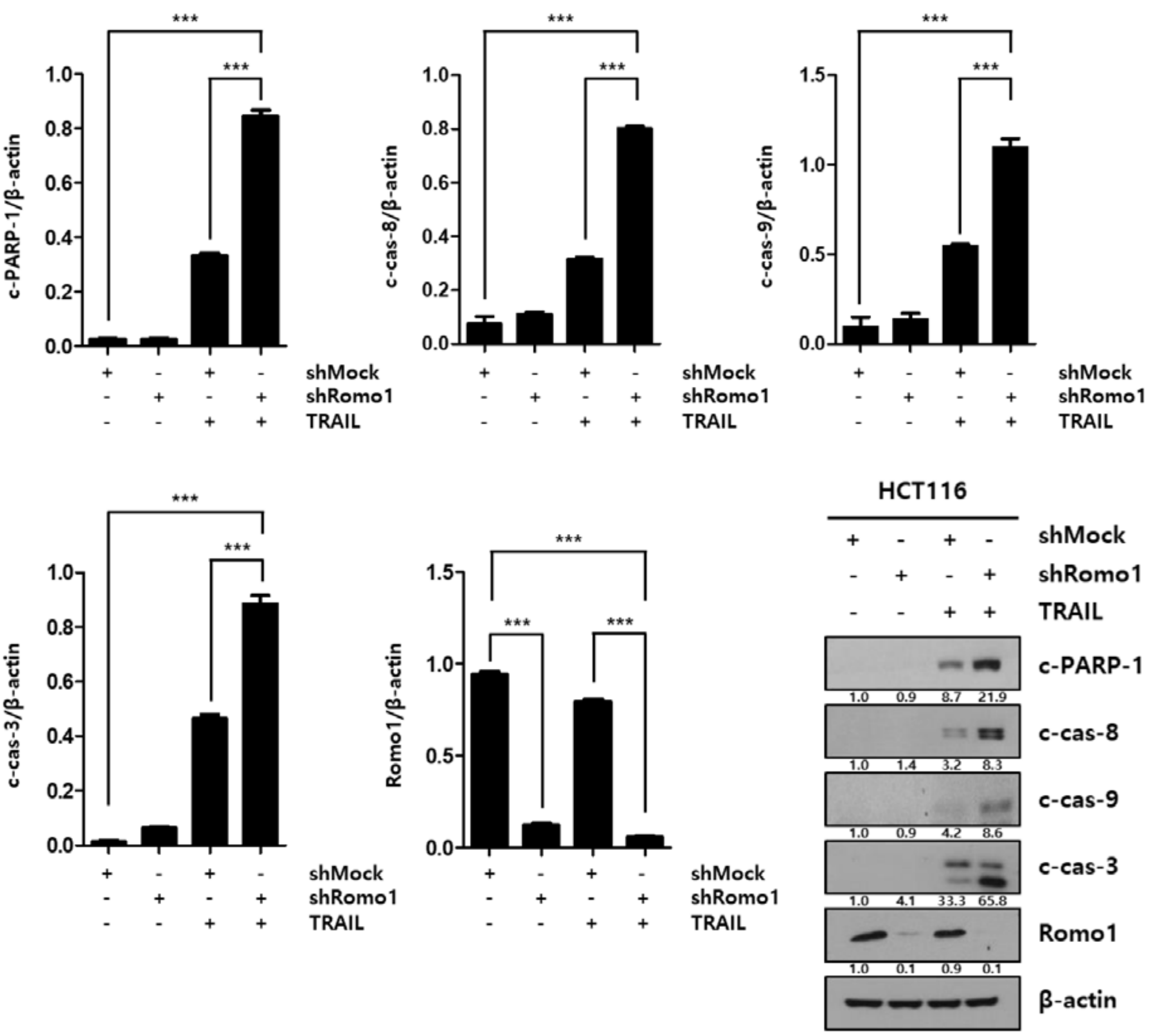

Figure S9. The bar graph represents the mean standard deviation (SD) of western blot band density of Figure 1E. *** stands for *** $p < 0.001$.

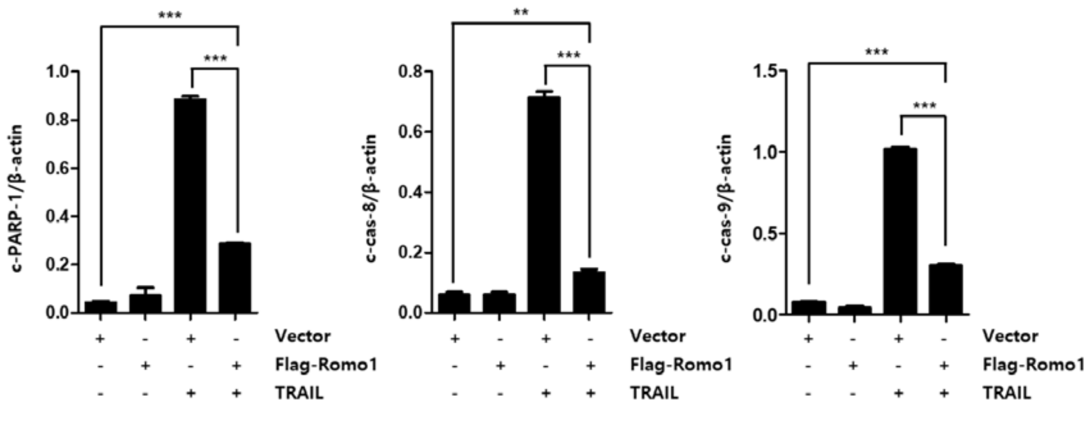

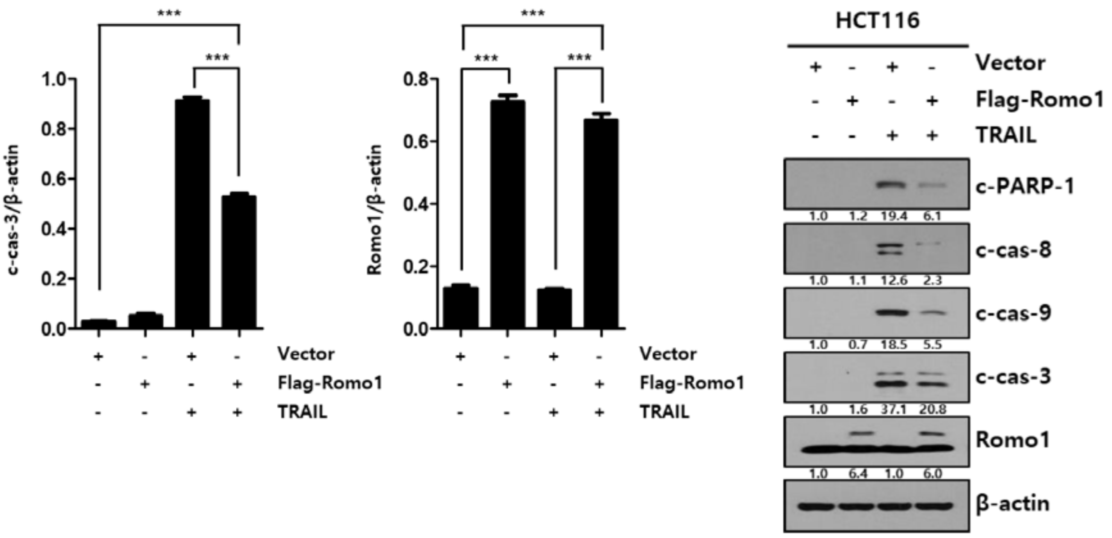

Figure S10. The bar graph represents the mean standard deviation (SD) of western blot band density of Figure 1F. **, *** stands for ** $p < 0.01$, and *** $p < 0.001$.

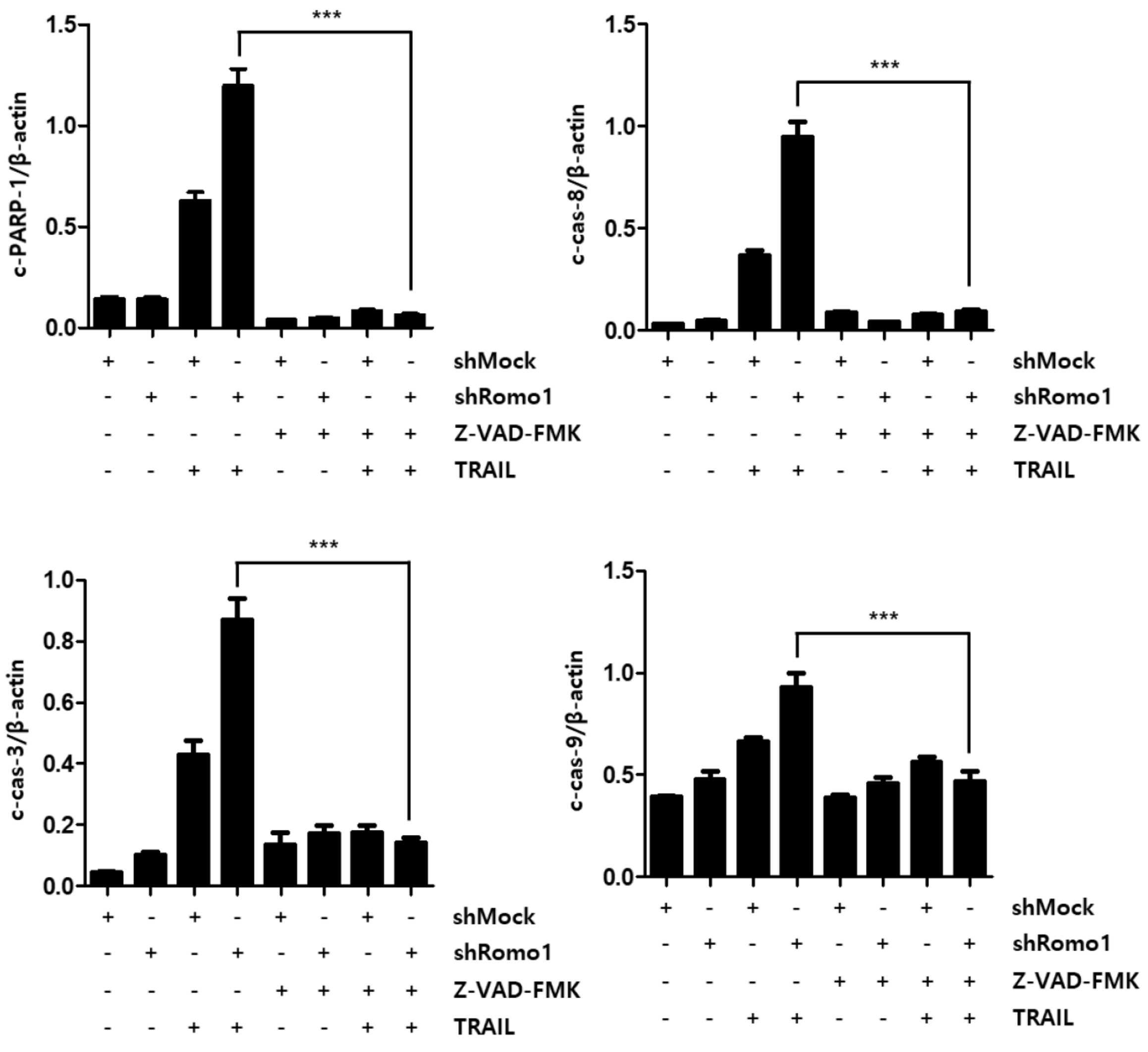

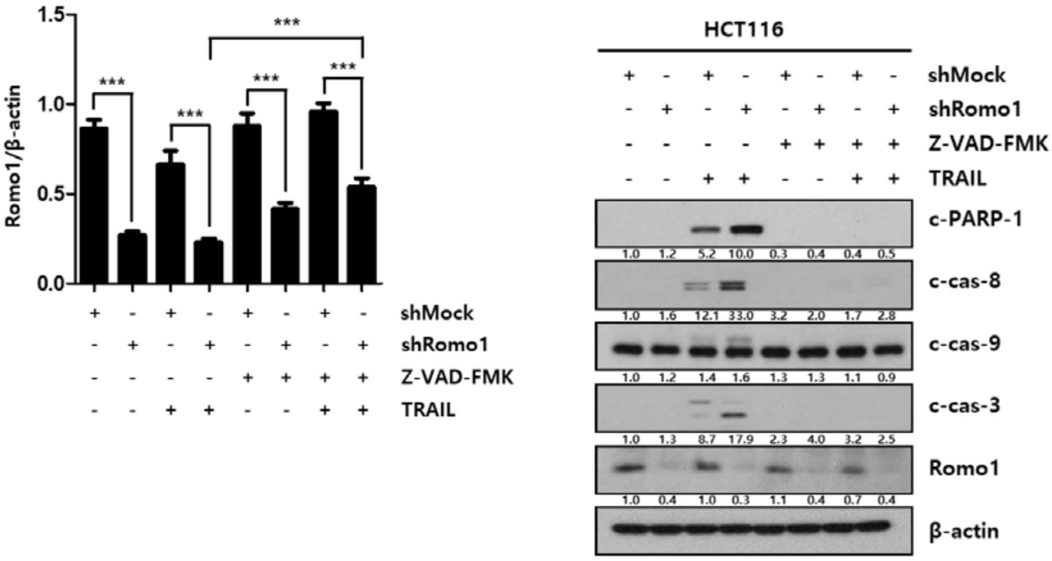

Figure S11. The bar graph represents the mean standard deviation (SD) of western blot band density of Figure 1G. *** $p < 0.001$.

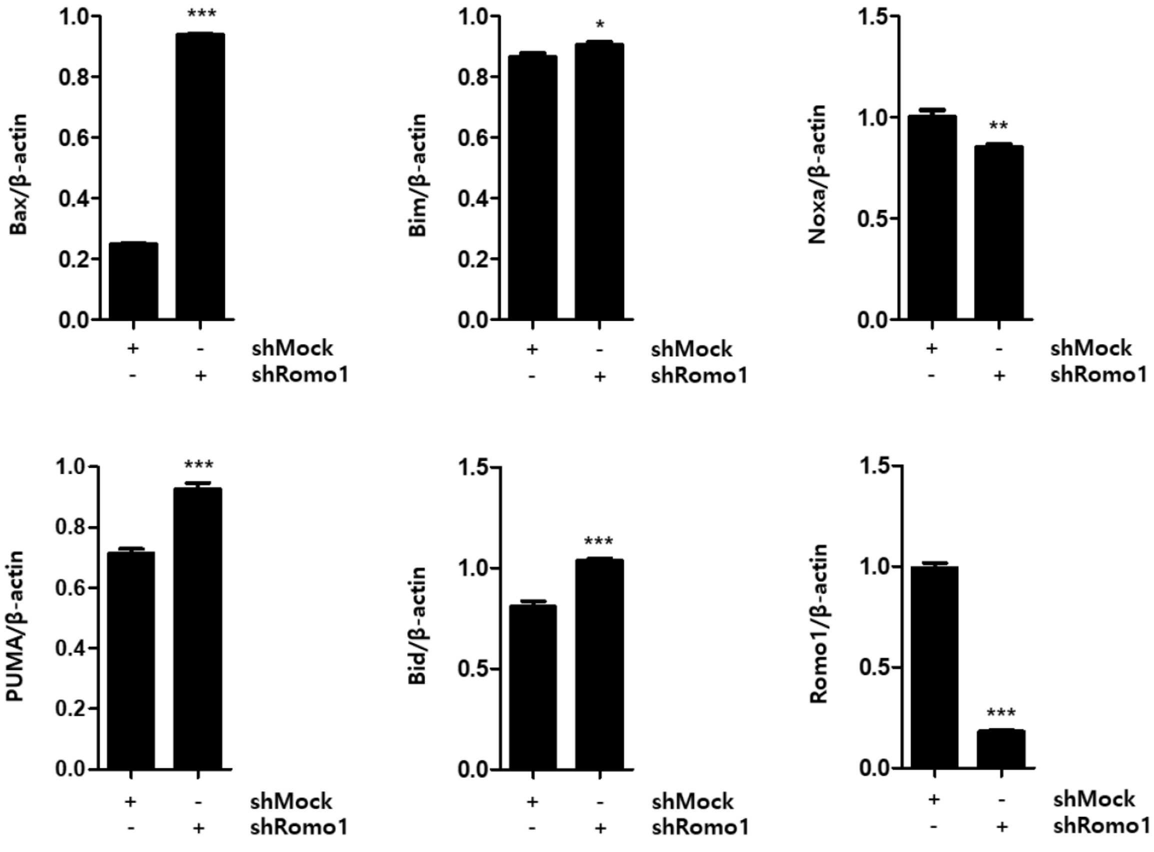

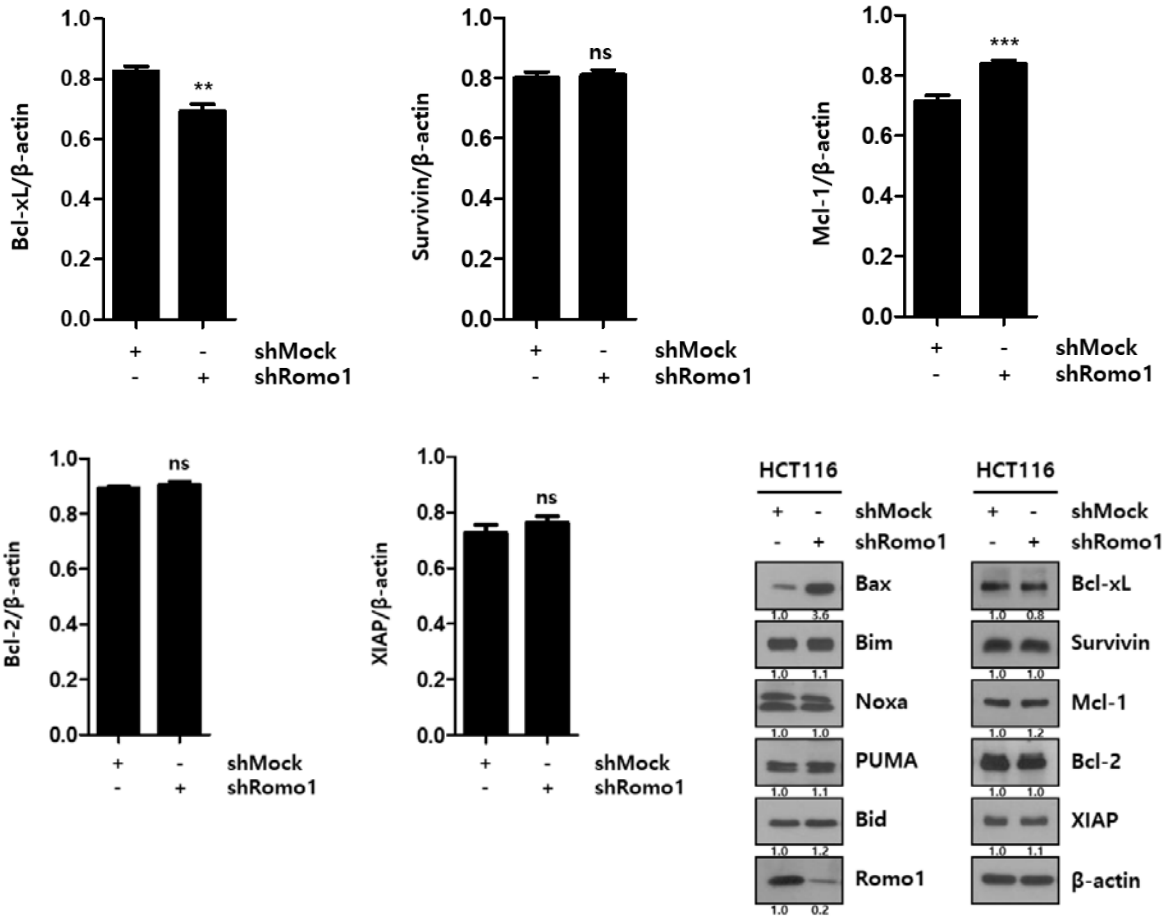

Figure S12. The bar graph represents the mean standard deviation (SD) of western blot band density of Figure 2A. *, **, *** stands for * $p < 0.05$, ** $p < 0.01$, and *** $p < 0.001$.

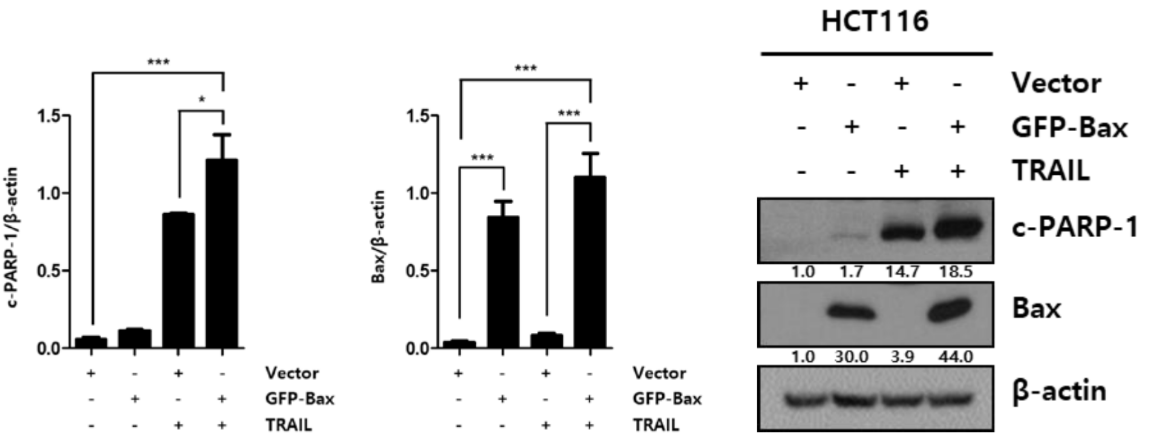

Figure S13. The bar graph represents the mean standard deviation (SD) of western blot band density of Figure 2C. *, *** stands for * $p < 0.05$, *** $p < 0.001$.

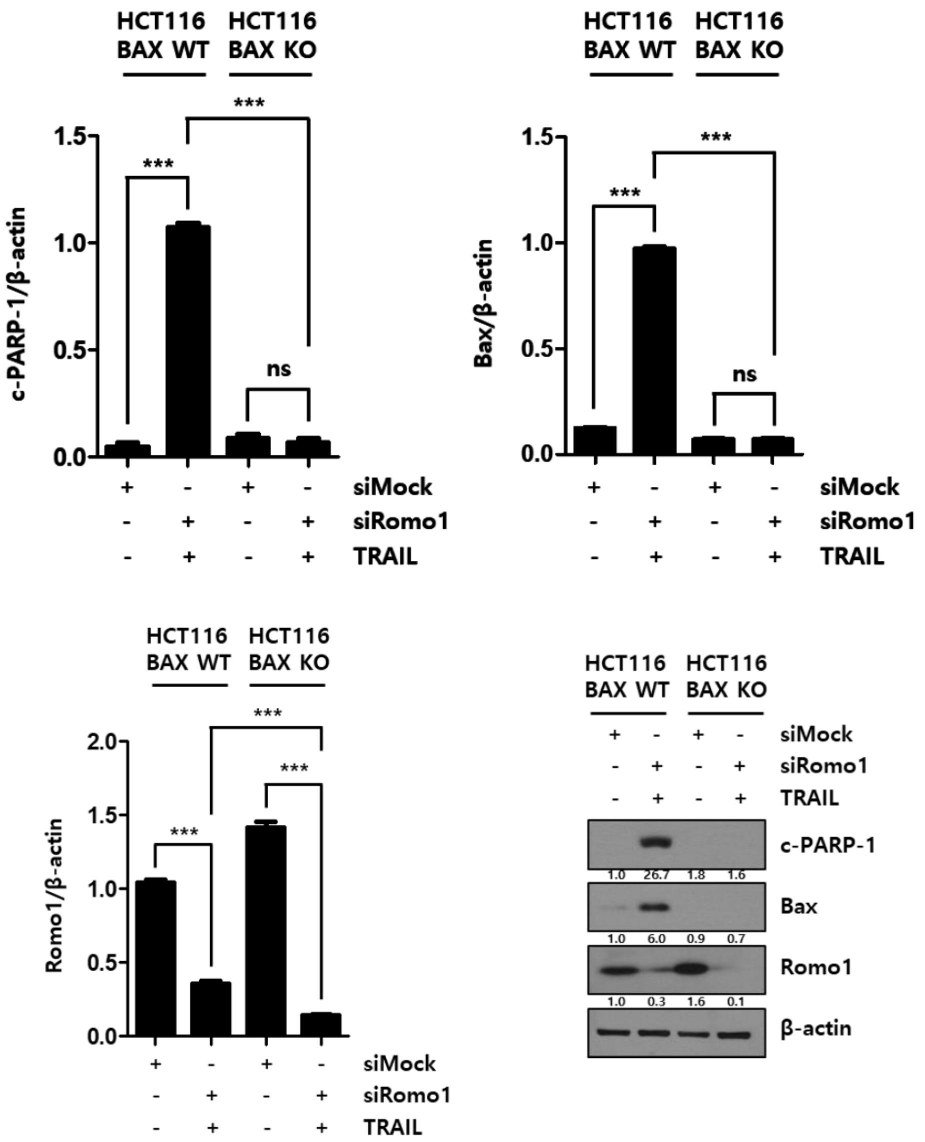

Figure S14. The bar graph represents the mean standard deviation (SD) of western blot band density of Figure 2D. *** stands for *** $p < 0.001$.

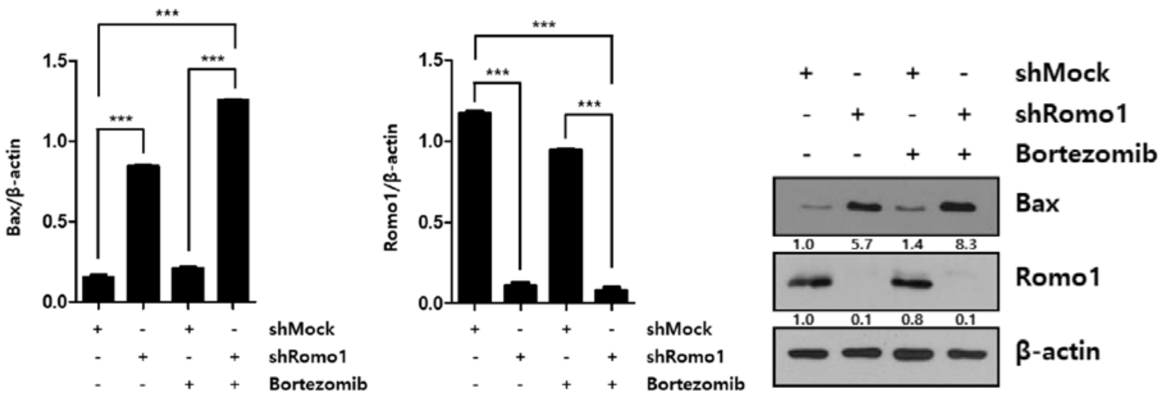

Figure S15. The bar graph represents the mean standard deviation (SD) of western blot band density of Figure 2E. *** stands for *** $p < 0.001$.

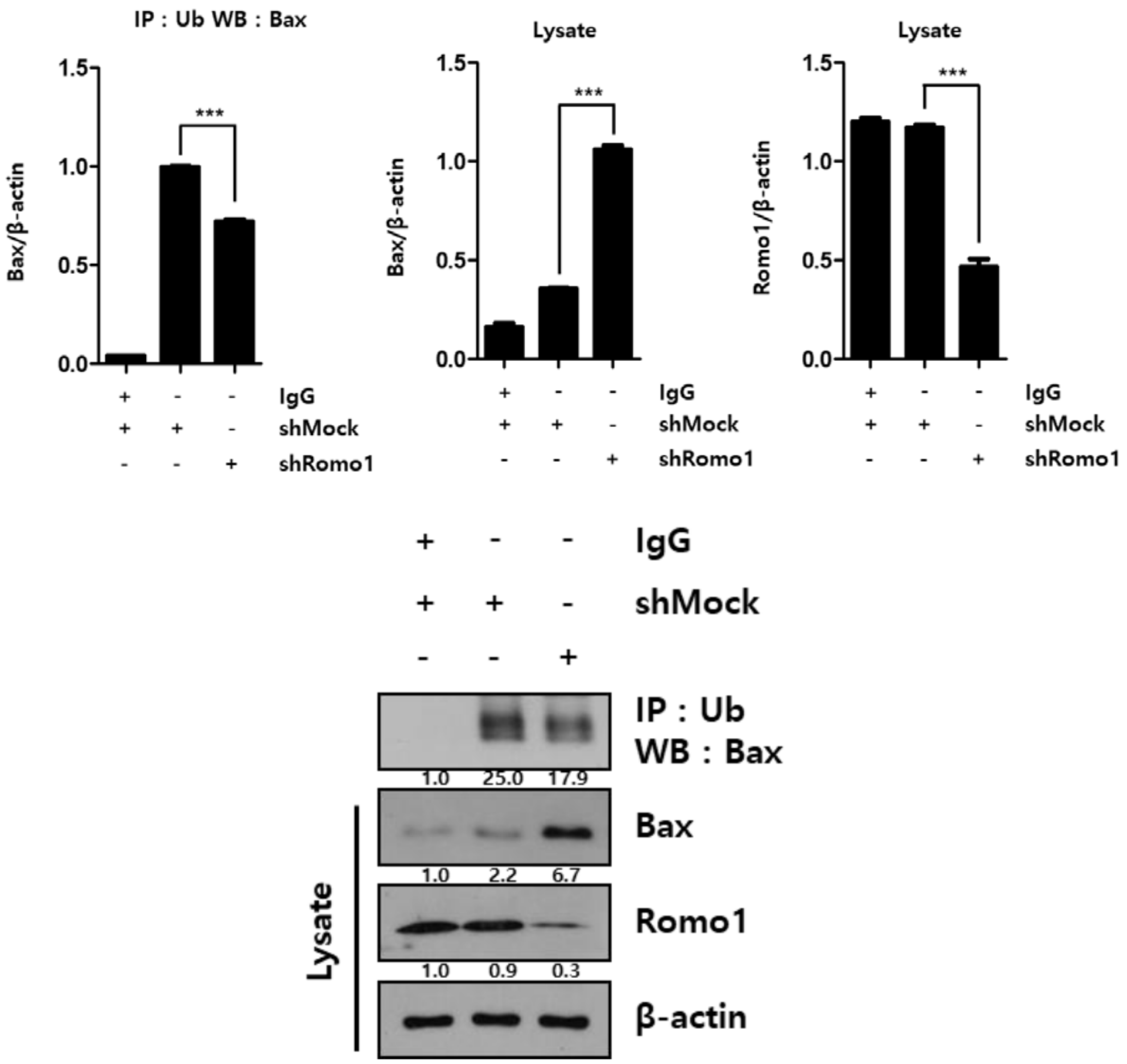

Figure S16. The bar graph represents the mean standard deviation (SD) of western blot band density of Figure 2F. *** stands for *** $p < 0.001$.

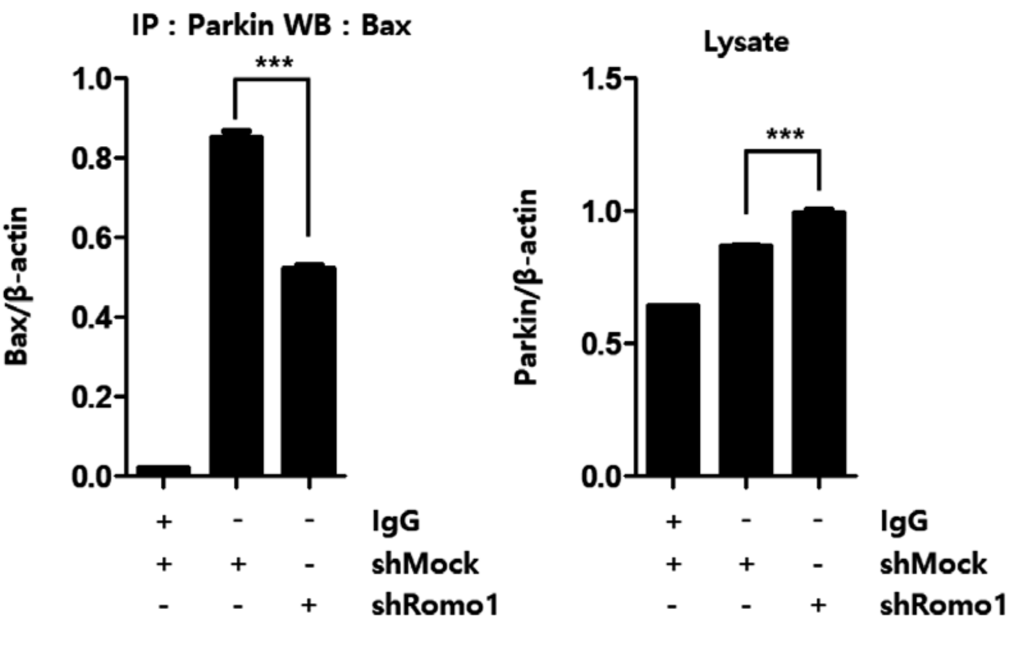

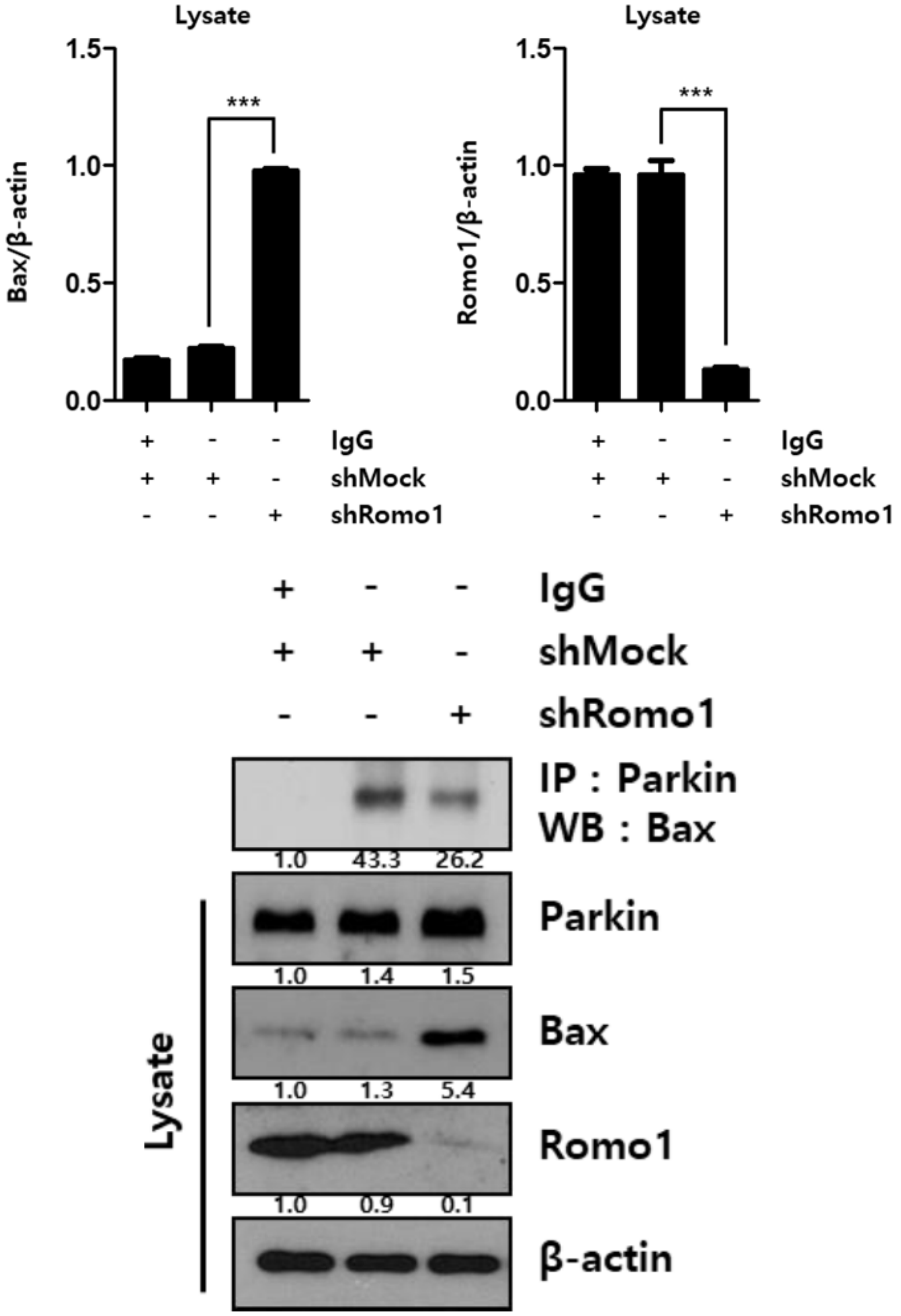

Figure S17. The bar graph represents the mean standard deviation (SD) of western blot band density of Figure 2G. *** stands for *** $p < 0.001$.

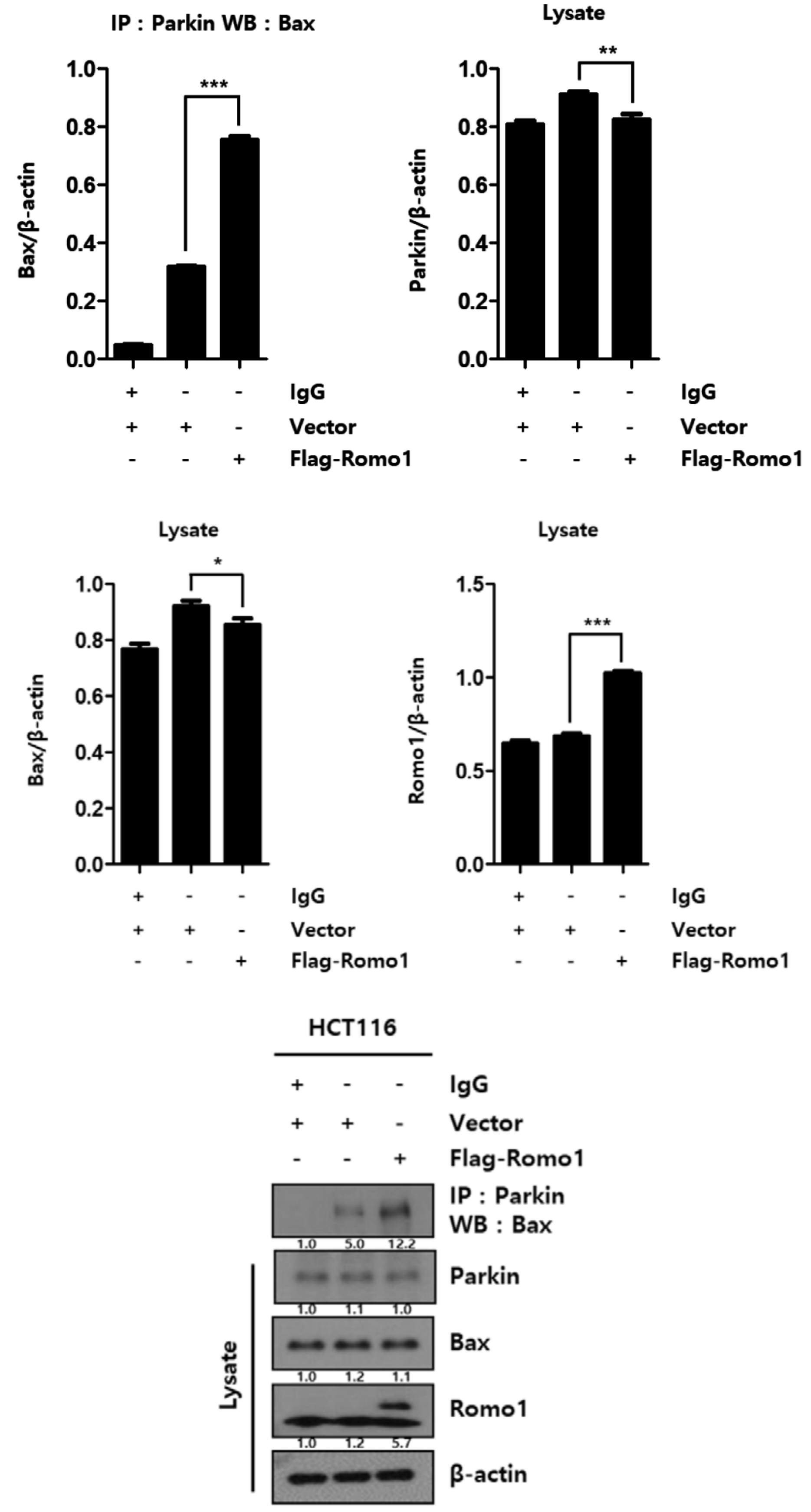

Figure S18. The bar graph represents the mean standard deviation (SD) of western blot band density of Figure 2H. *, **, *** stands for * $p < 0.05$, ** $p < 0.01$, and *** $p < 0.001$.

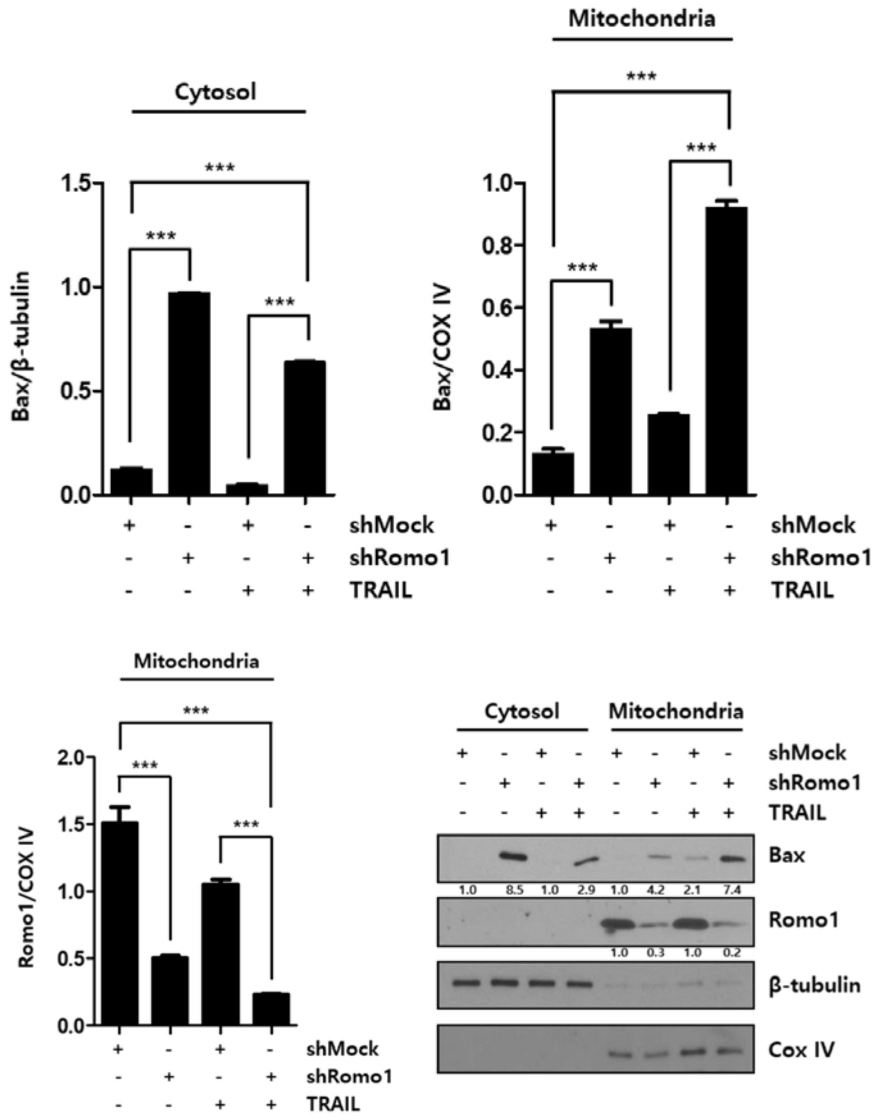

Figure S19. The bar graph represents the mean standard deviation (SD) of western blot band density of Figure 3A. *** stands for *** $p < 0.001$.

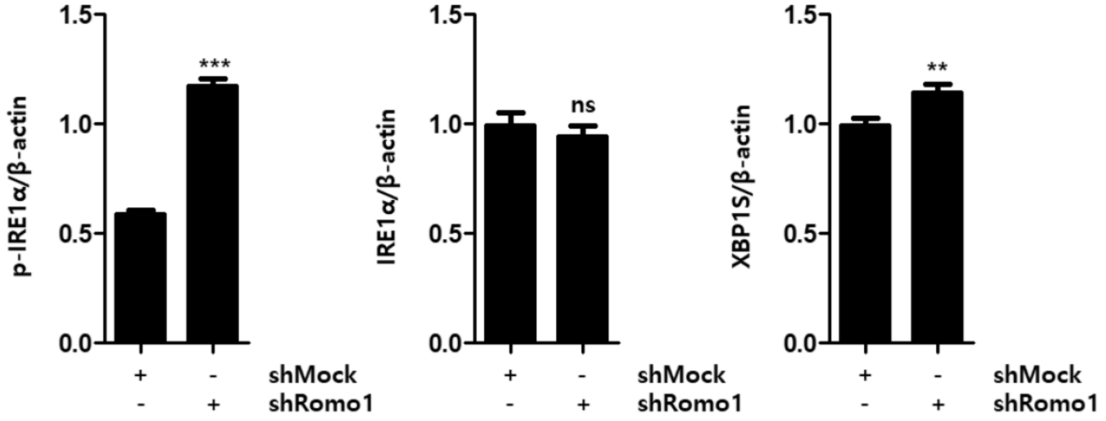

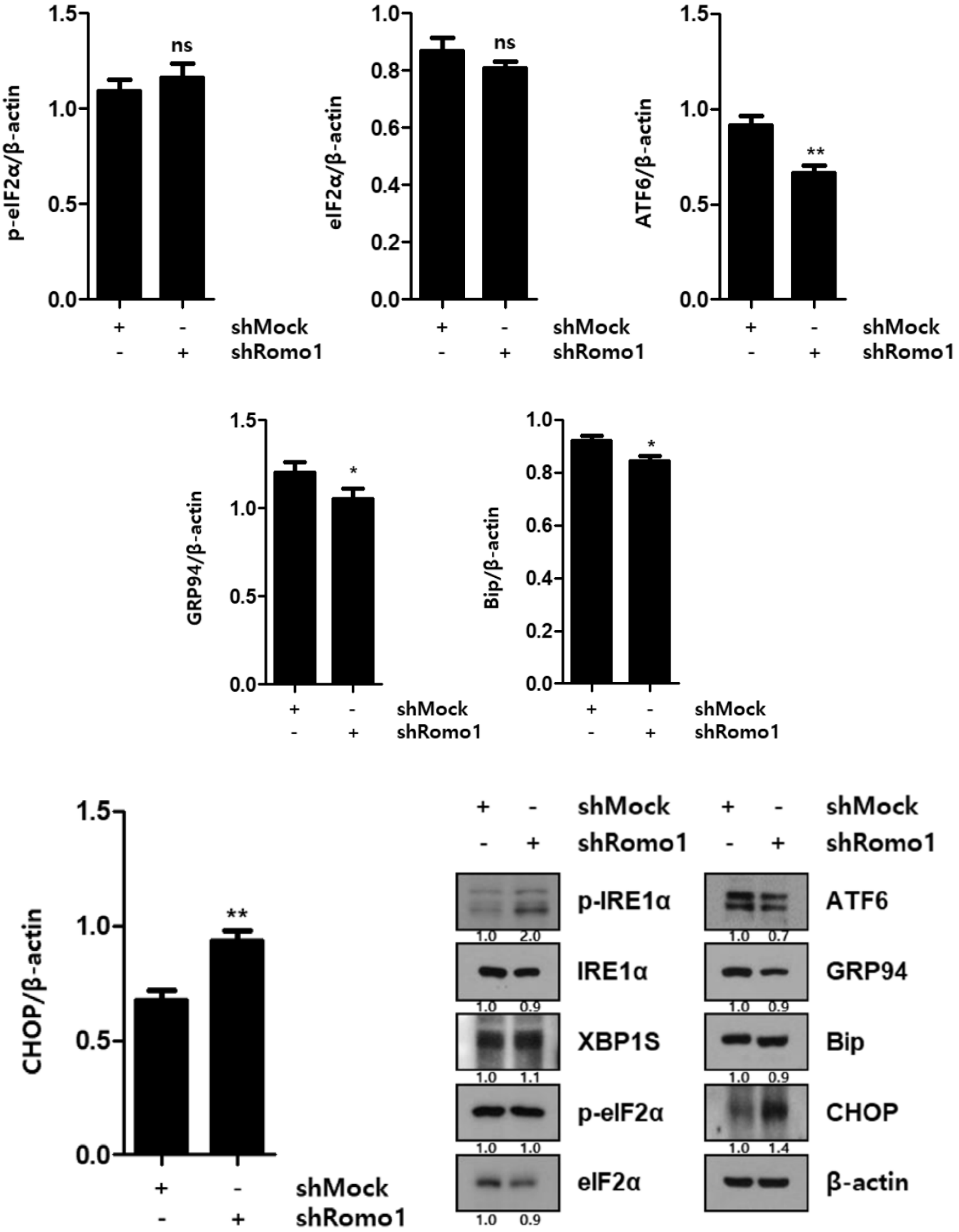

Figure S20. The bar graph represents the mean standard deviation (SD) of western blot band density of Figure 4A. *, **, *** stands for * $p < 0.05$, ** $p < 0.01$, and *** $p < 0.001$.

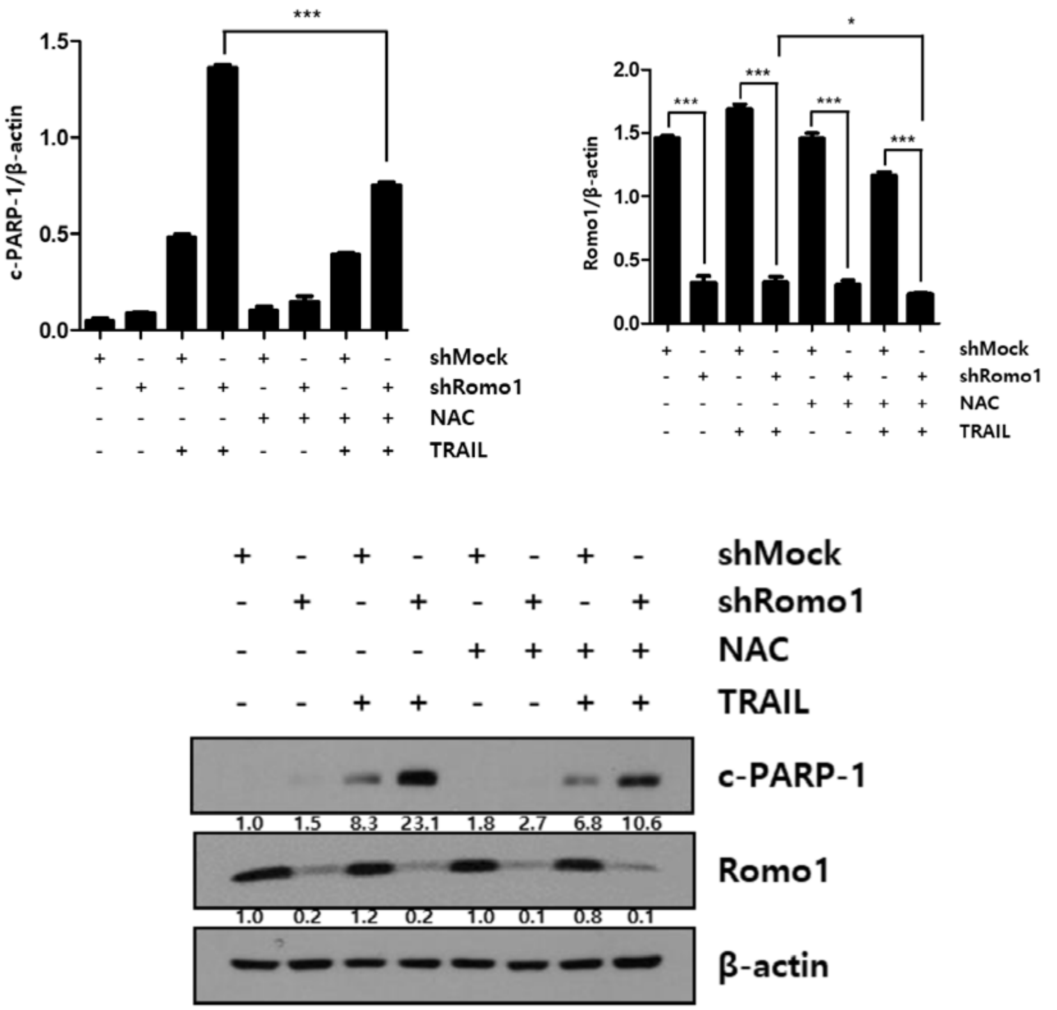

Figure S21. The bar graph represents the mean standard deviation (SD) of western blot band density of Figure 4D. *, *** stands for * $p < 0.05$, *** $p < 0.001$.

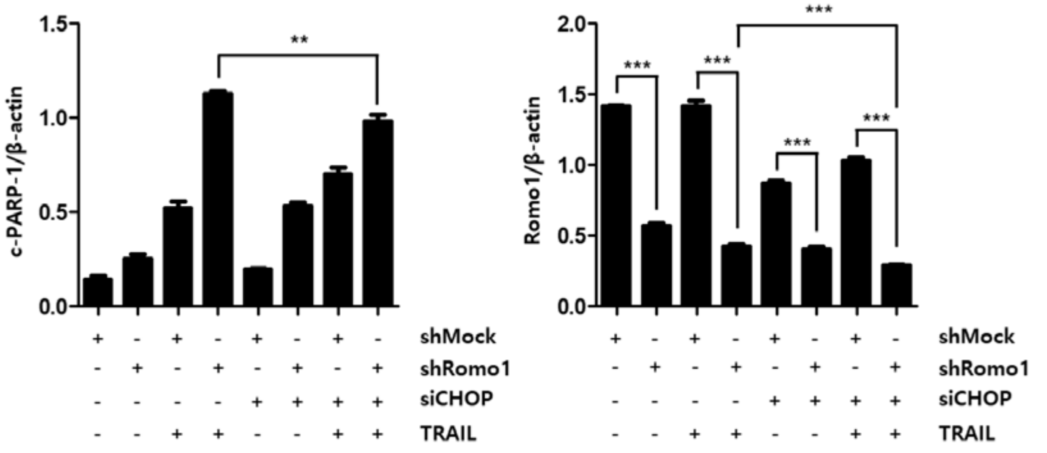

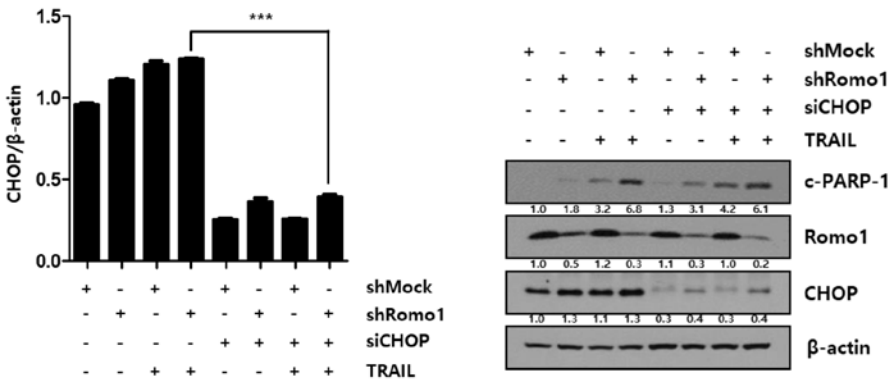

Figure S22. The bar graph represents the mean standard deviation (SD) of western blot band density of Figure 4E. **, *** stands for ** $p < 0.01$, *** $p < 0.001$.

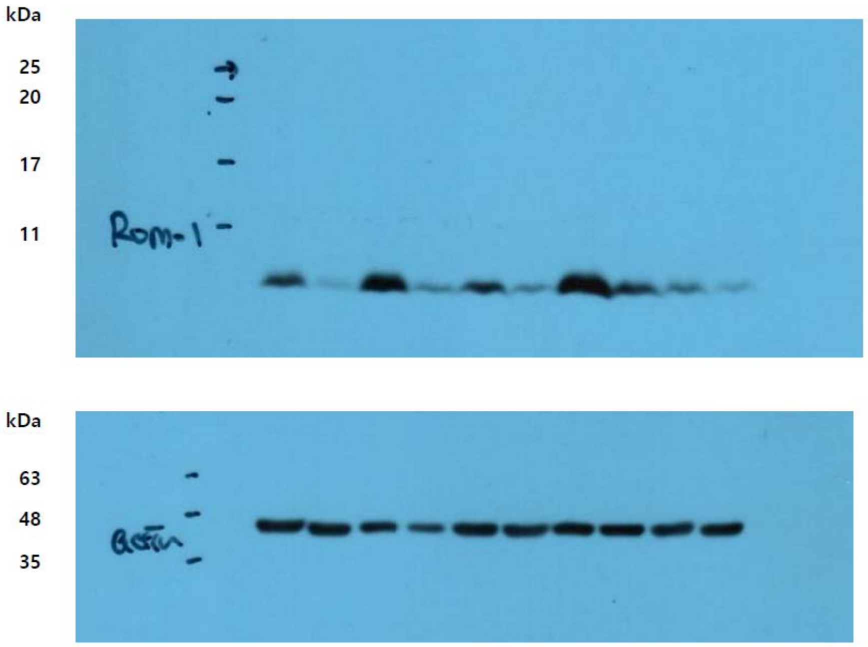

Figure S23. Whole Blots for Western Blot analysis for Figure 1C.

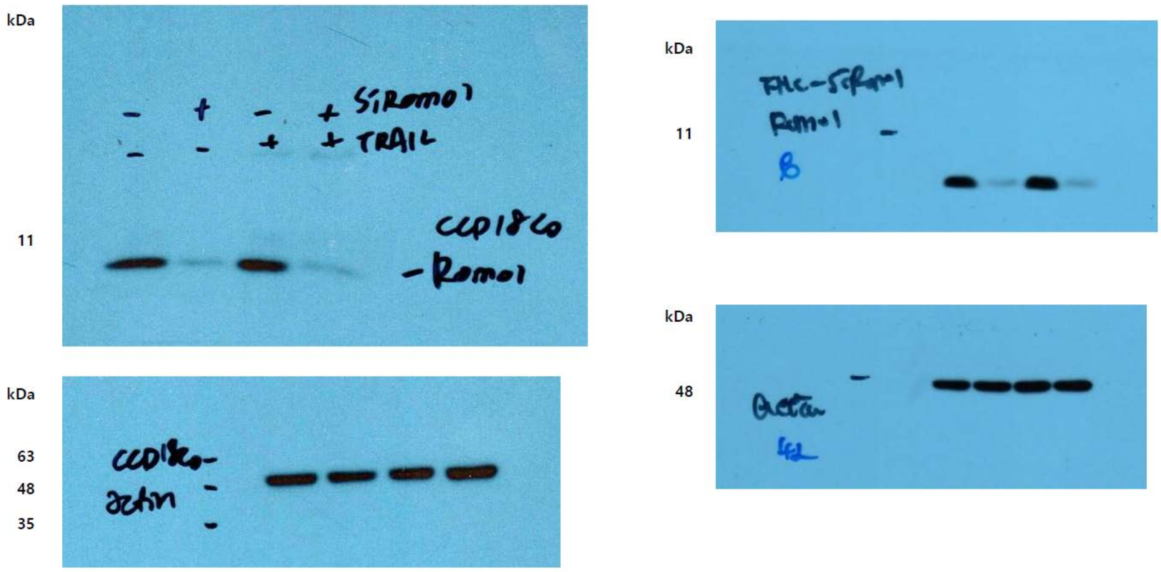

Figure S24. Whole Blots for Western Blot analysis for Figure 1D.

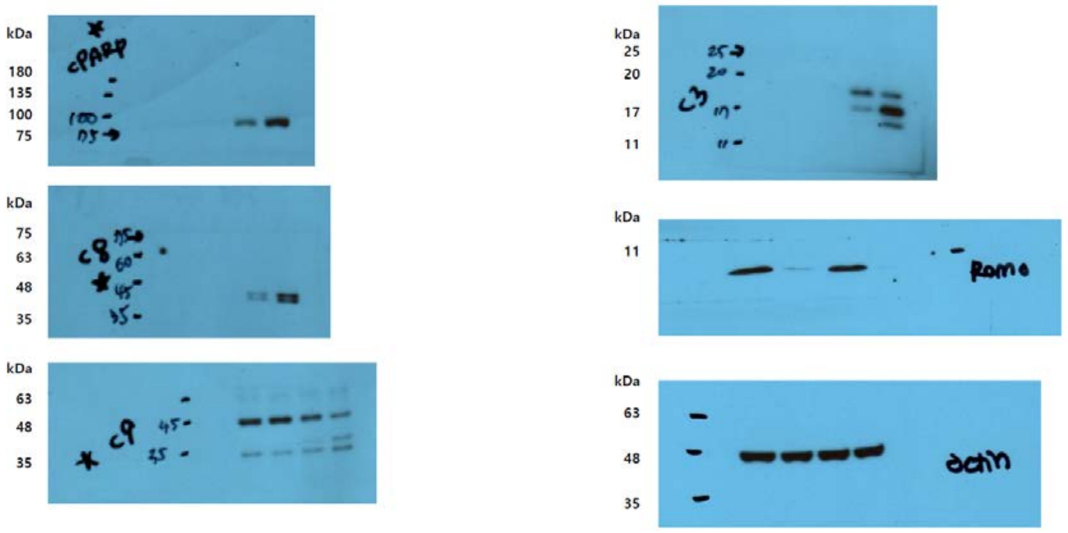

Figure S25. Whole Blots for Western Blot analysis for Figure 1E.

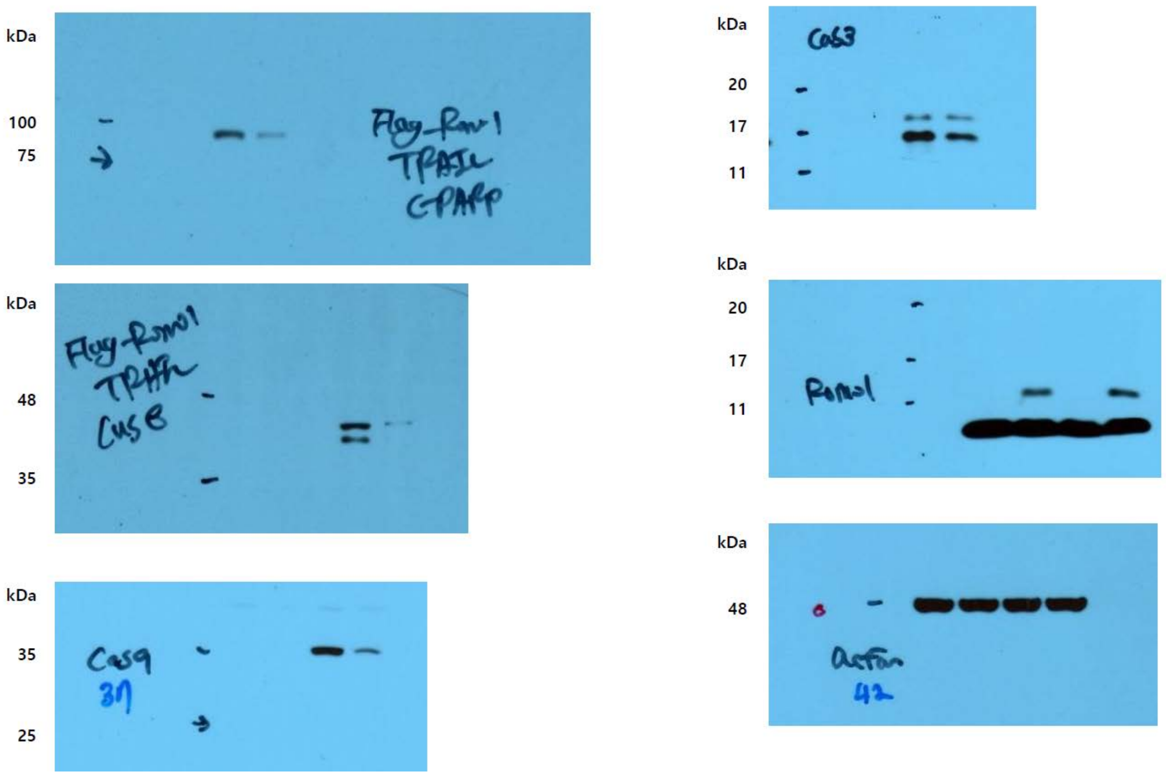

Figure S26. Whole Blots for Western Blot analysis for Figure 1F.

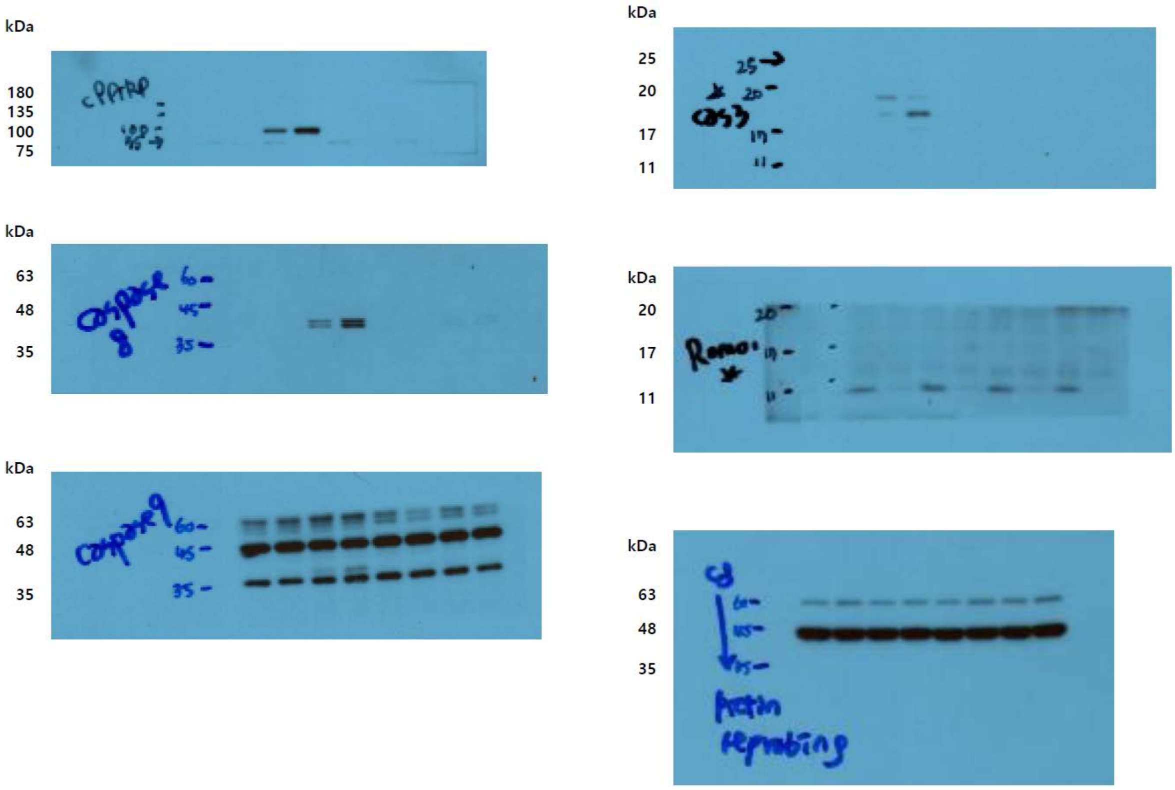

Figure S27. Whole Blots for Western Blot analysis for Figure 1G.

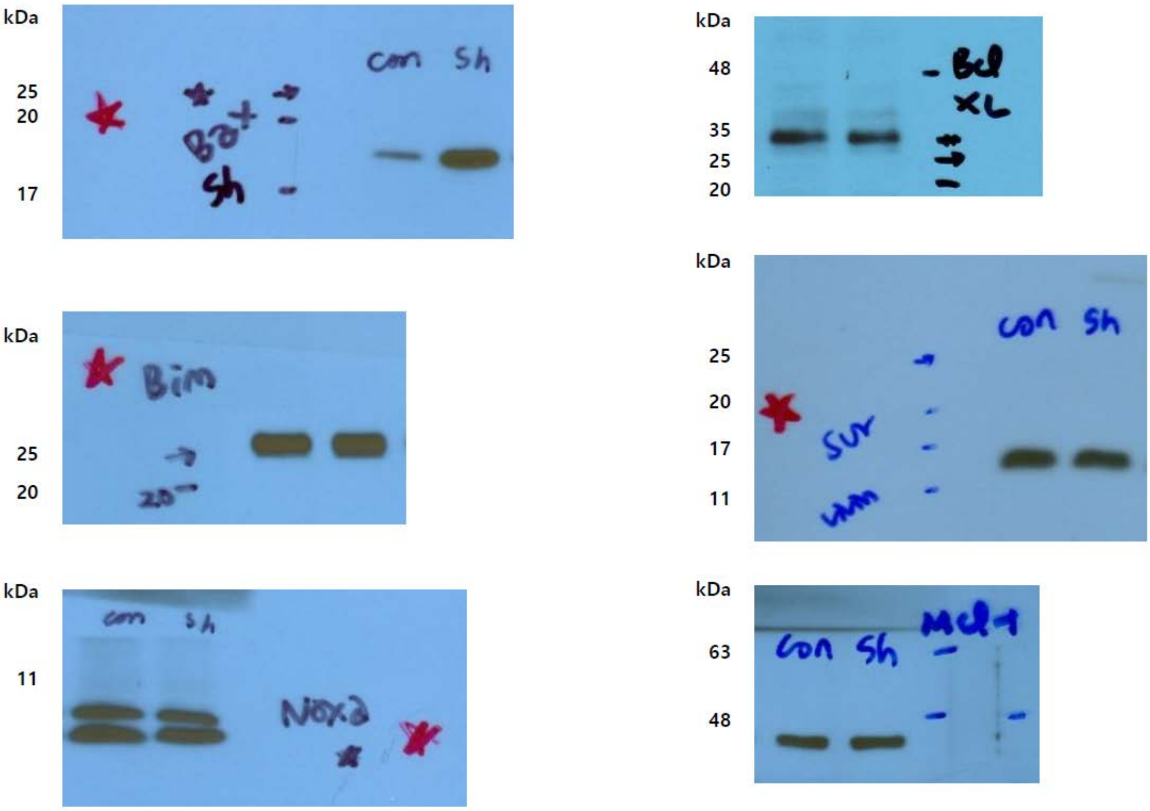

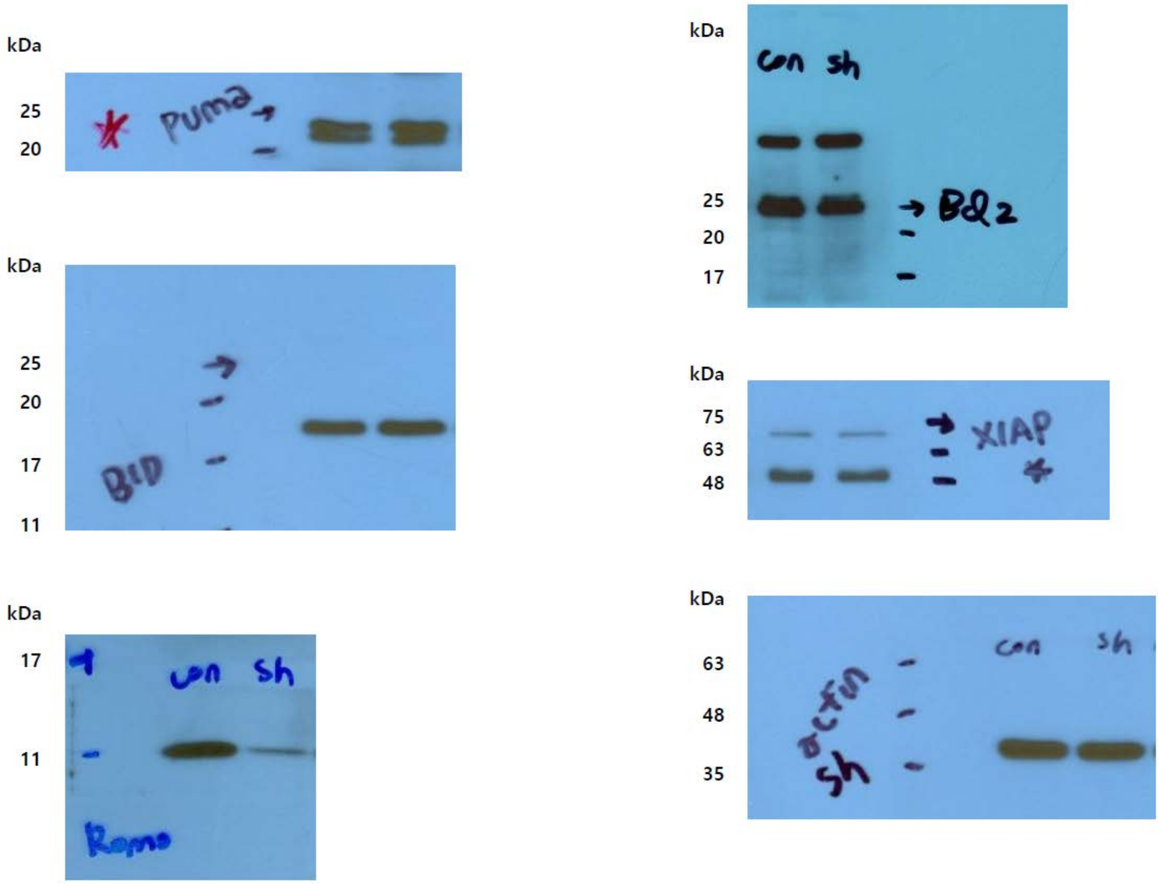

Figure S28. Whole Blots for Western Blot analysis for Figure 2A.

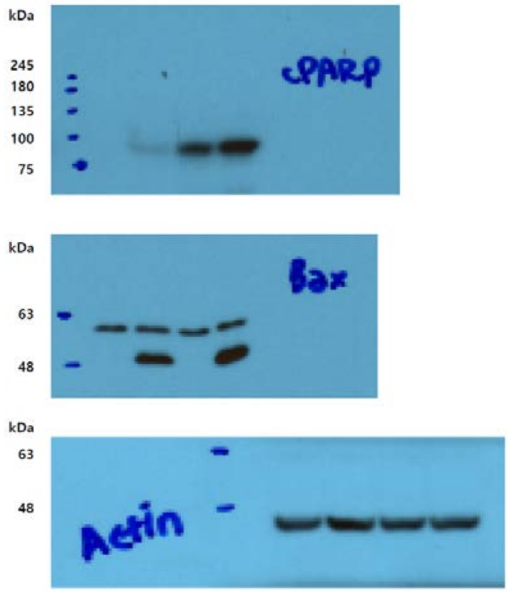

Figure S29. Whole Blots for Western Blot analysis for Figure 2C.

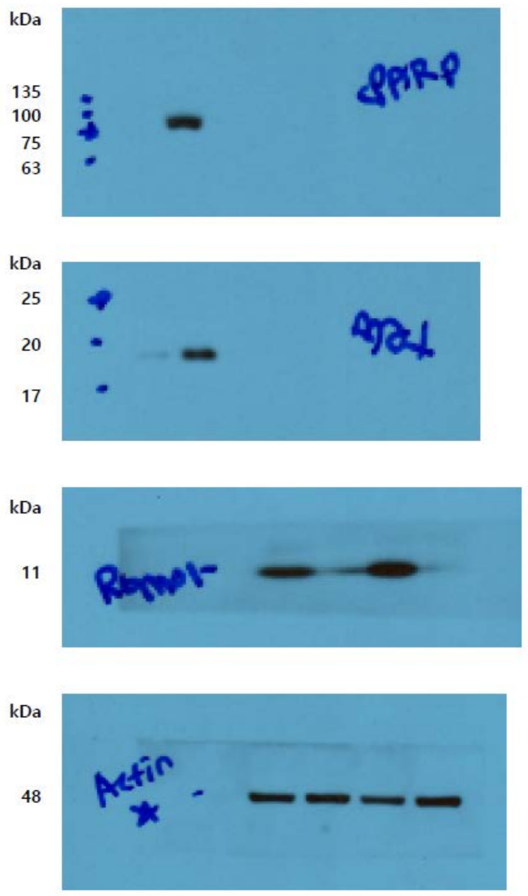

Figure S30. Whole Blots for Western Blot analysis for Figure 2D.

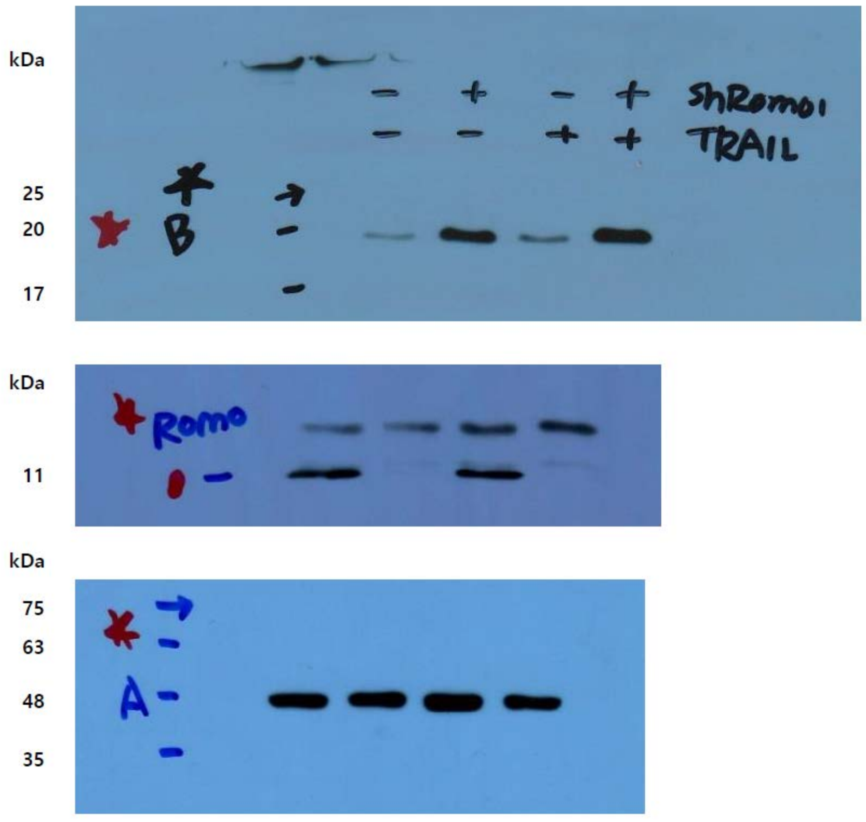

Figure S31. Whole Blots for Western Blot analysis for Figure 2E.

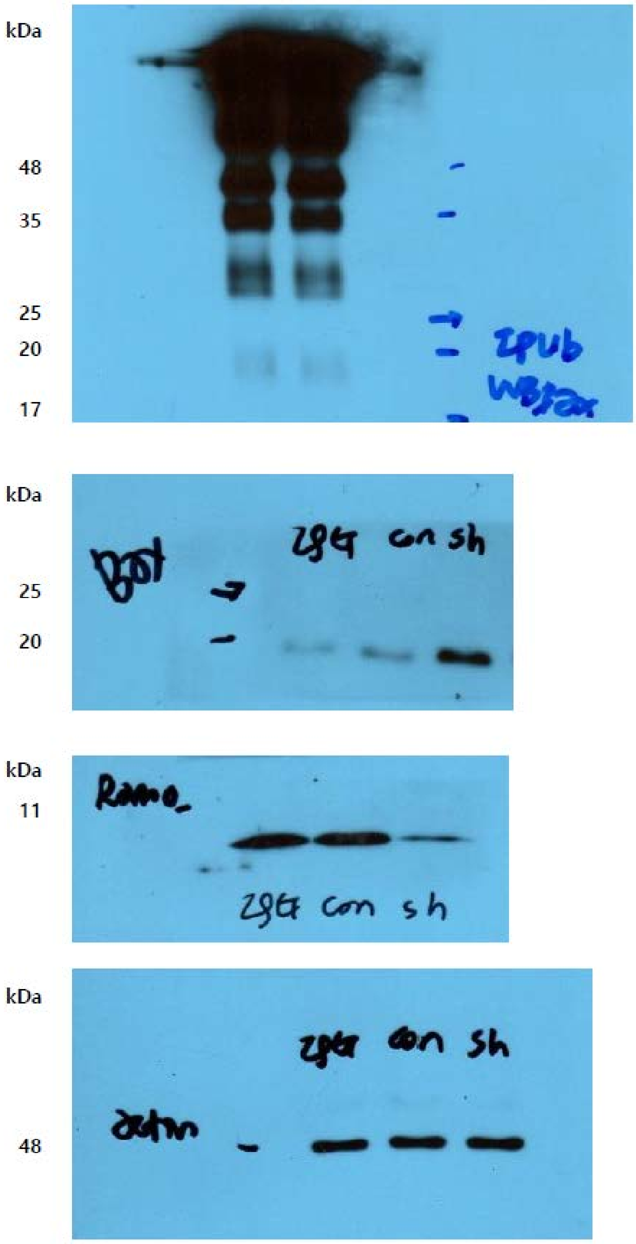

Figure S32. Whole Blots for Western Blot analysis for Figure 2F.

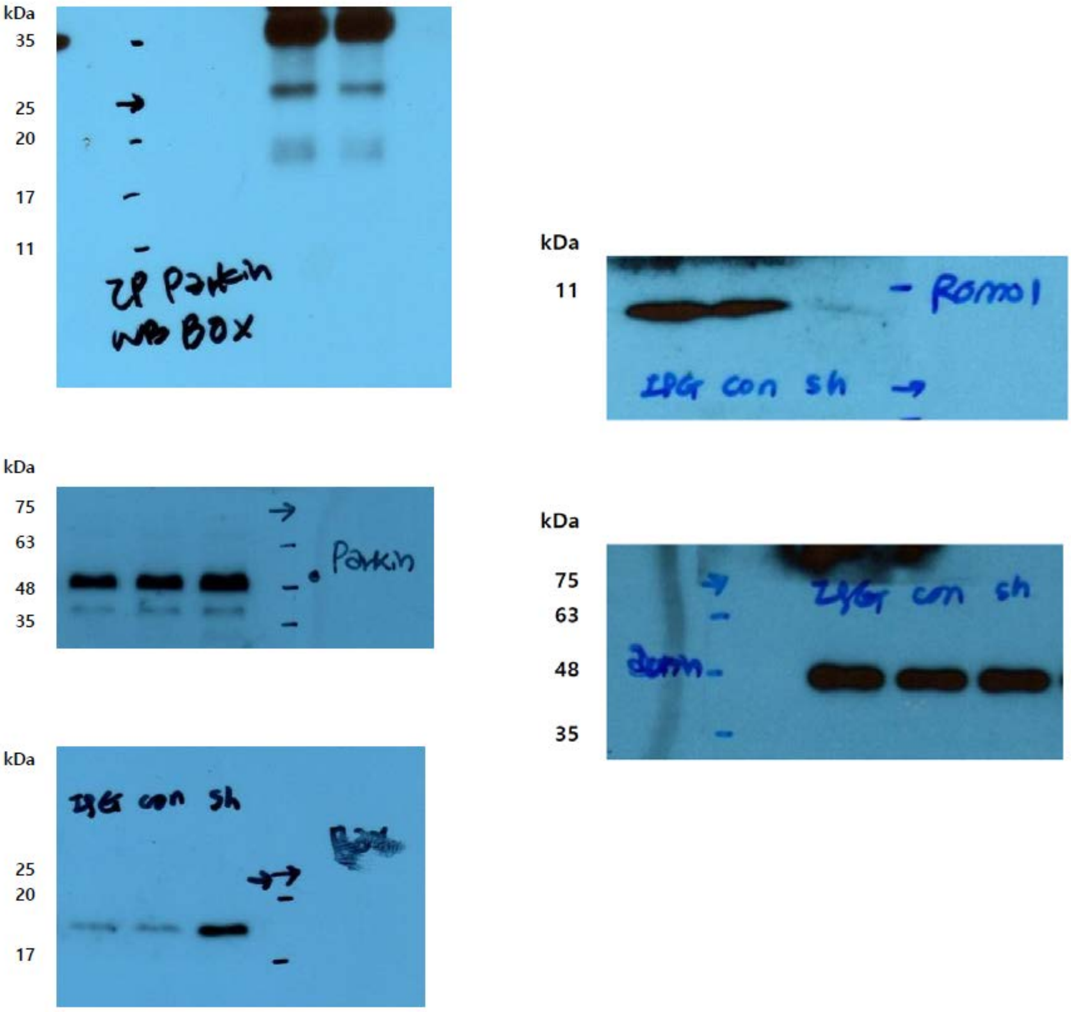

Figure S33. Whole Blots for Western Blot analysis for Figure 2G.

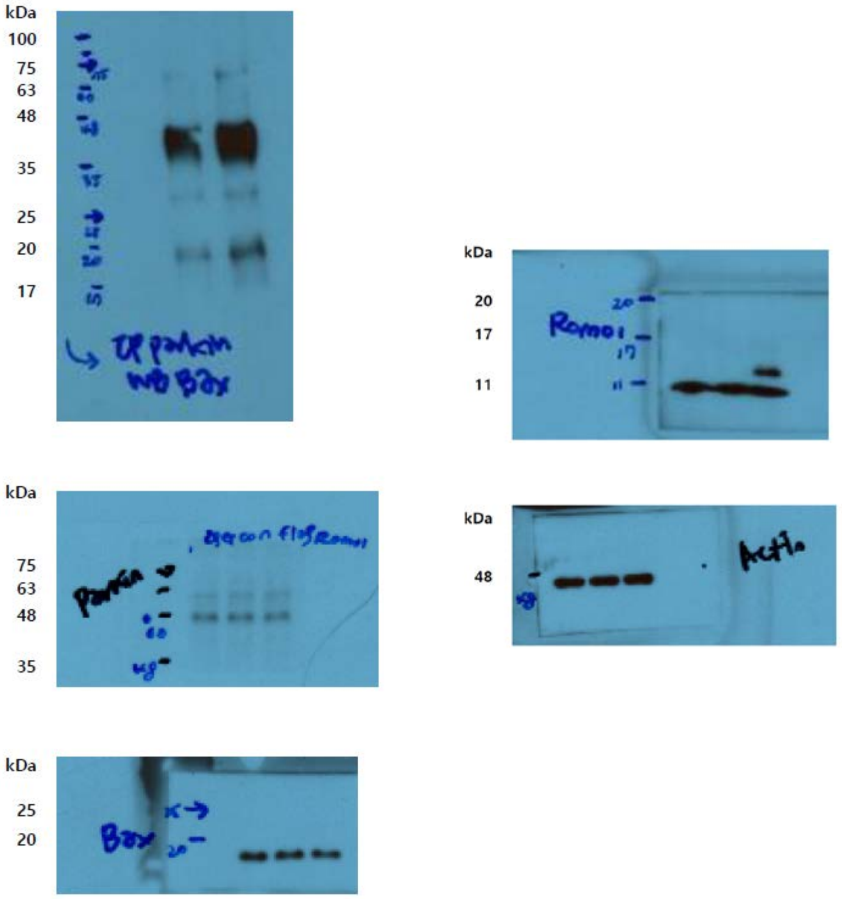

Figure S34. Whole Blots for Western Blot analysis for Figure 2H.

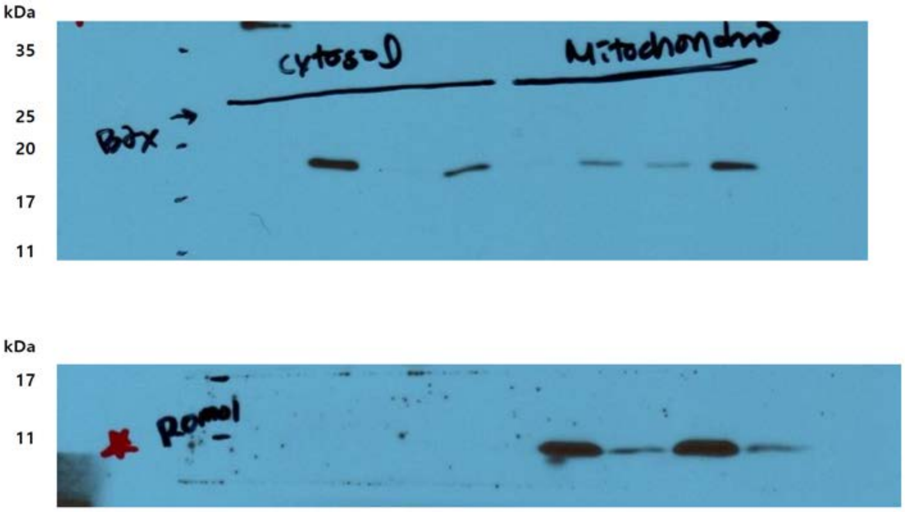

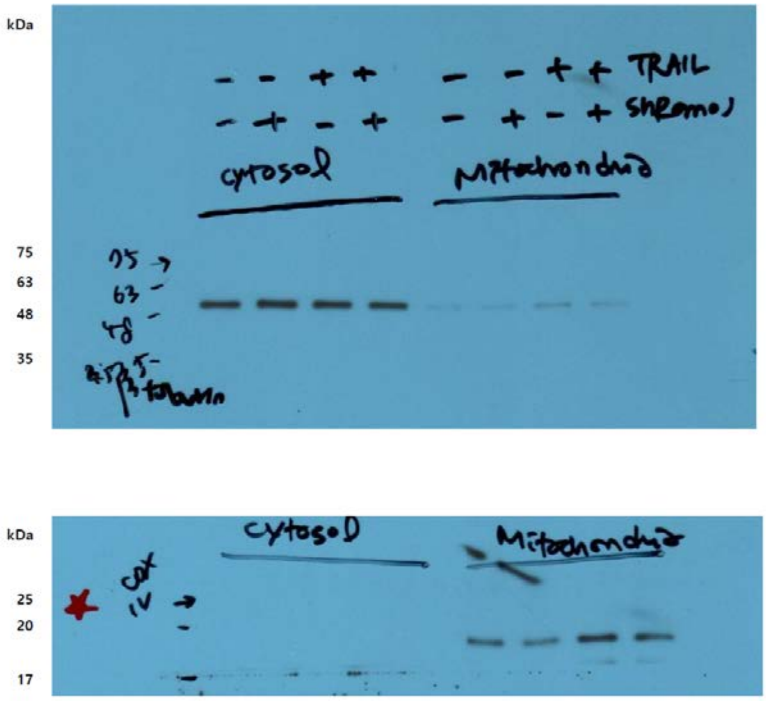

Figure S35. Whole Blots for Western Blot analysis for Figure 3A.

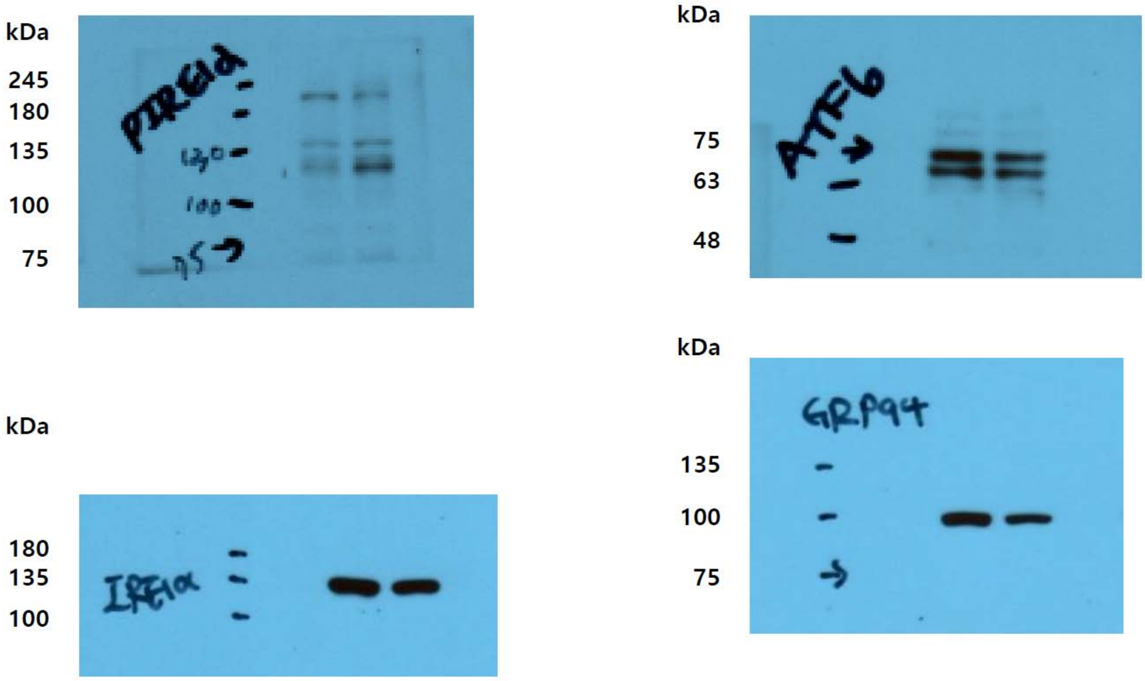

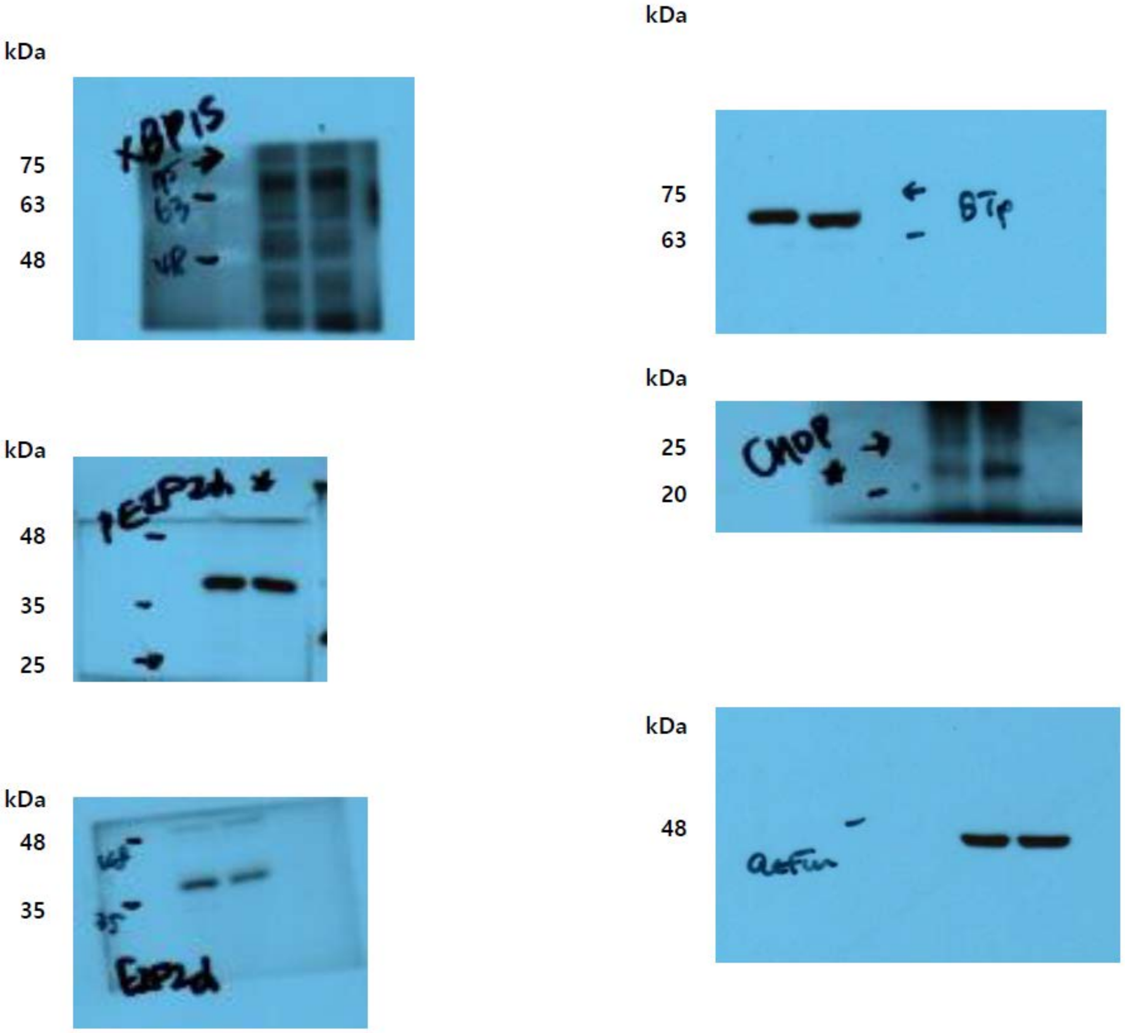

Figure S36. Whole Blots for Western Blot analysis for Figure 4A.

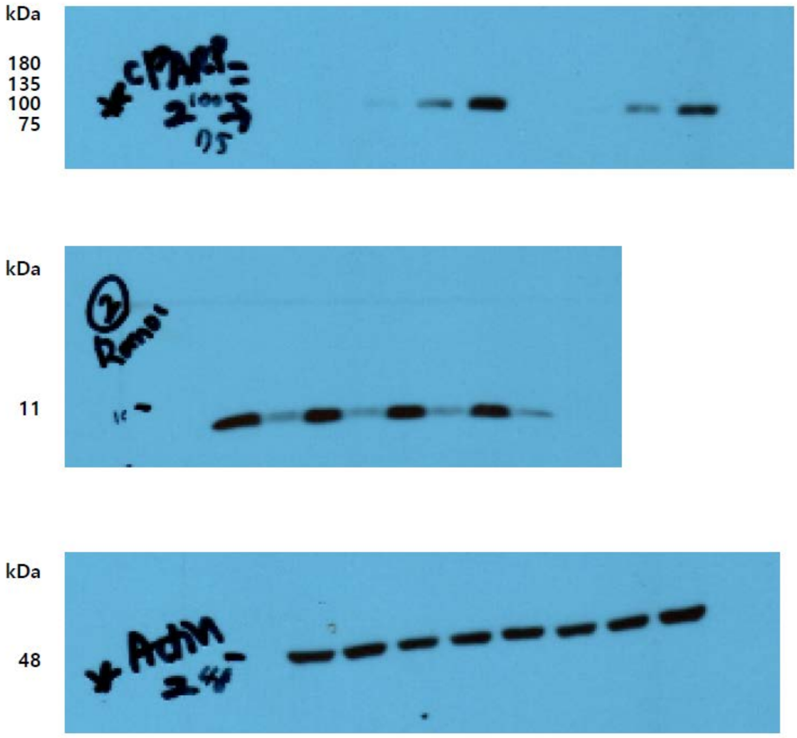

Figure S37. Whole Blots for Western Blot analysis for Figure 4D.

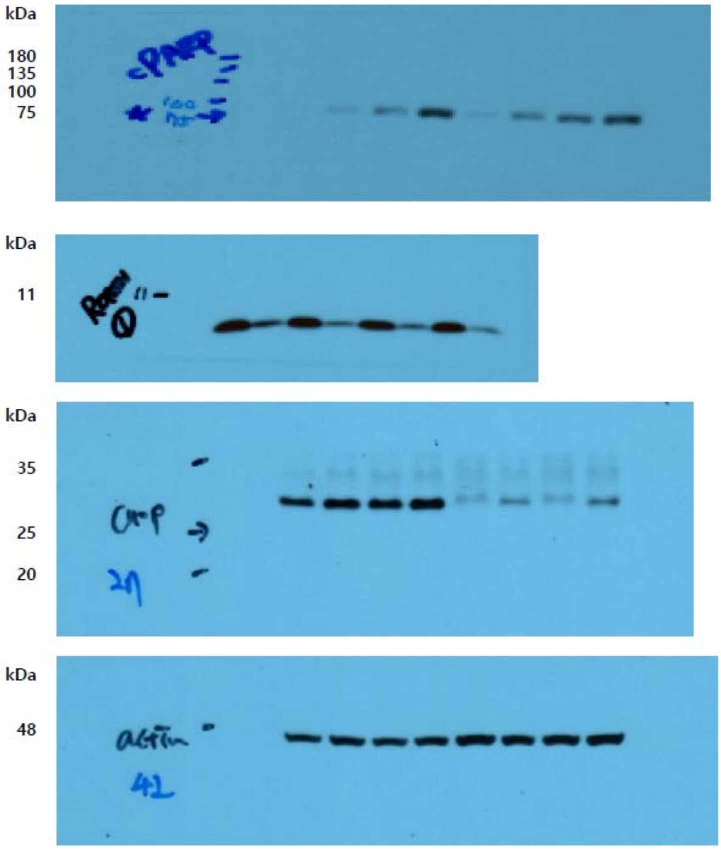

Figure S38. Whole Blots for Western Blot analysis for Figure 4E.

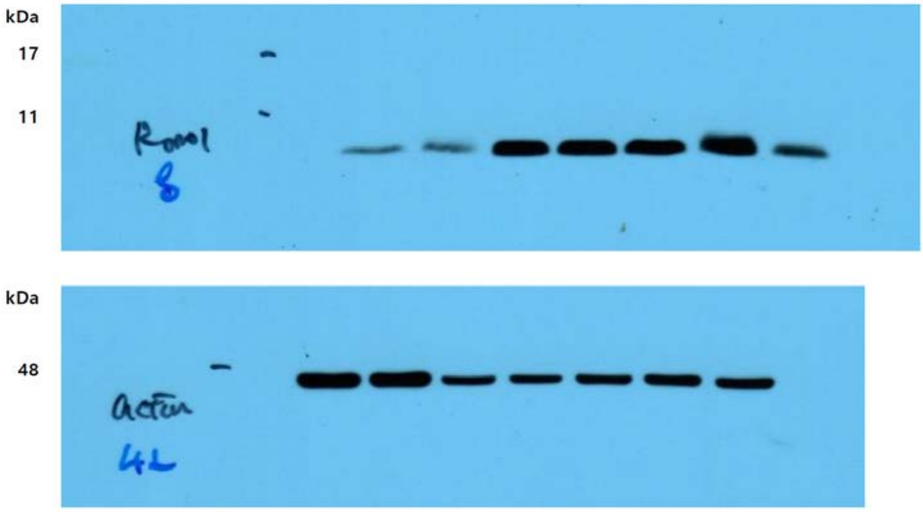

Figure S39. Whole Blots for Western Blot analysis for Figure S1.

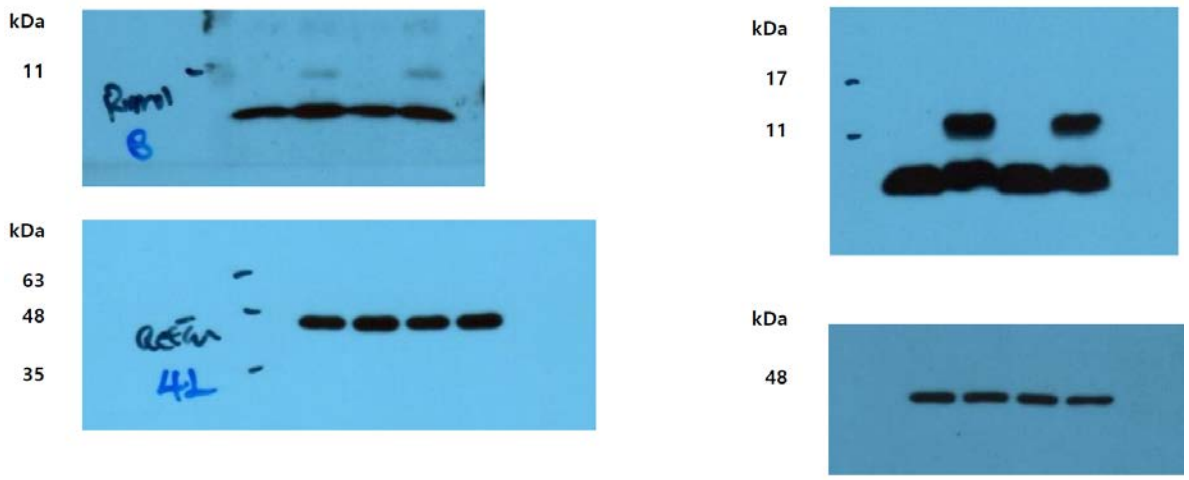

Figure S40. Whole Blots for Western Blot analysis for Figure S3.

© 2020 by the authors. Licensee MDPI, Basel, Switzerland. This article is an open access article distributed under the terms and conditions of the Creative Commons Attribution (CC BY) license (http://creativecommons.org/licenses/by/4.0/).
